# Factors associated with non-fatal heart failure and atrial fibrillation or flutter within the first 30 days post COPD exacerbation: a nested case-control study

**DOI:** 10.1186/s12890-024-03035-4

**Published:** 2024-05-04

**Authors:** Emily L. Graul, Clementine Nordon, Kirsty Rhodes, Shruti Menon, Mahmoud Al Ammouri, Constantinos Kallis, Anne E. Ioannides, Hannah R. Whittaker, Nicholas S. Peters, Jennifer K. Quint

**Affiliations:** 1 School of Public Health, Imperial College London, 86 Wood Lane, London, W12 0BZ UK; 2 Biopharmaceuticals Medical, AstraZeneca, Academy House, 136 Hills Rd, Cambridge, CB2 8PA UK; 3 Medical and Scientific Affairs, AstraZeneca, 2 Pancras Sq, London, N1C 4AG UK; 4 National Heart and Lung Institute, Imperial College London, 86 Wood Lane, London, W12 0BZ UK

**Keywords:** Chronic Obstructive Pulmonary Disease Exacerbations, Cardiovascular Disease, Heart Failure, Arrhythmia, Atrial Fibrillation, Flutter, Electronic Health Records, Epidemiology

## Abstract

**Background:**

An immediate, temporal risk of heart failure and arrhythmias after a Chronic Obstructive Pulmonary Disease (COPD) exacerbation has been demonstrated, particularly in the first month post-exacerbation. However, the clinical profile of patients who develop heart failure (HF) or atrial fibrillation/flutter (AF) following exacerbation is unclear. Therefore we examined factors associated with people being hospitalized for HF or AF, respectively, following a COPD exacerbation.

**Methods:**

We conducted two nested case-control studies, using primary care electronic healthcare records from the Clinical Practice Research Datalink Aurum linked to Hospital Episode Statistics, Office for National Statistics for mortality, and socioeconomic data (2014-2020). Cases had hospitalization for HF or AF within 30 days of a COPD exacerbation, with controls matched by GP practice (HF 2:1;AF 3:1). We used conditional logistic regression to explore demographic and clinical factors associated with HF and AF hospitalization.

**Results:**

Odds of HF hospitalization (1,569 cases, 3,138 controls) increased with age, type II diabetes, obesity, HF and arrhythmia history, exacerbation severity (hospitalization), most cardiovascular medications, GOLD airflow obstruction, MRC dyspnea score, and chronic kidney disease. Strongest associations were for severe exacerbations (adjusted odds ratio (aOR)=6.25, 95%CI 5.10-7.66), prior HF (aOR=2.57, 95%CI 1.73-3.83), age≥80 years (aOR=2.41, 95%CI 1.88-3.09), and prior diuretics prescription (aOR=2.81, 95%CI 2.29-3.45).

Odds of AF hospitalization (841 cases, 2,523 controls) increased with age, male sex, severe exacerbation, arrhythmia and pulmonary hypertension history and most cardiovascular medications. Strongest associations were for severe exacerbations (aOR=5.78, 95%CI 4.45-7.50), age≥80 years (aOR=3.15, 95%CI 2.26-4.40), arrhythmia (aOR=3.55, 95%CI 2.53-4.98), pulmonary hypertension (aOR=3.05, 95%CI 1.21-7.68), and prescription of anticoagulants (aOR=3.81, 95%CI 2.57-5.64), positive inotropes (aOR=2.29, 95%CI 1.41-3.74) and anti-arrhythmic drugs (aOR=2.14, 95%CI 1.10-4.15).

**Conclusions:**

Cardiopulmonary factors were associated with hospitalization for HF in the 30 days following a COPD exacerbation, while only cardiovascular-related factors and exacerbation severity were associated with AF hospitalization. Understanding factors will help target people for prevention.

**Supplementary Information:**

The online version contains supplementary material available at 10.1186/s12890-024-03035-4.

## Background

Exacerbations of Chronic Obstructive Pulmonary Disease (COPD) are associated with an increased risk of cardiovascular disease (CVD) [1] likely due to linked pathophysiology, hypoxic state, and an amplified systemic inflammatory response [2, 3]. Previous studies have demonstrated an increased, immediate period of risk for hospitalized cardiovascular events following a COPD exacerbation compared with non-exacerbating patients, [1, 4] with the risk highest within the first month and following a severe (hospitalized) exacerbation [1, 5, 6]. The 30 day risk of arrhythmias and heart failure (HF) following an exacerbation, [6–8] approach [4, 6] or even surpass [7, 9] 3-fold, compared to those who did not have an exacerbation.

Globally, the prevalence of HF in COPD is high, [10] and, of patients hospitalized for exacerbation, 20% have existing, undertreated HF [11]. Incident HF attributed to exacerbations is thought to come from the increase in pulmonary arterial pressures, low blood oxygen levels [12] and activation of adrenoceptors of the autonomic nervous system [12, 13]. However, shared symptomology of HF and COPD exacerbations makes new HF diagnosis difficult, with HF often missed [3, 12, 14] or occurring in tandem; approximately 8% of people primarily diagnosed with HF also have a secondary diagnosis of exacerbation [15]. Studies have investigated COPD progression in HF [12] and conversely, HF progression across COPD phenotypes, [12, 16] but few have focused on exacerbating COPD alone [15] and no studies have examined factors associated with hospitalization with a HF diagnosis following a COPD exacerbation.

Arrhythmias are also common among people with COPD, with prevalence ranging from 5 to 15% globally, [10] and atrial fibrillation (AF) is the most common arrhythmia [17]. AF diagnoses at the time of an exacerbation are also common, with estimates around 17% [18], but due to shared, nonspecific symptoms between AF and COPD, differential diagnosis can be difficult [19]. During a COPD exacerbation, changes in blood gases from hypoxia and rising pulmonary pressure can lead to electrophysiological and structural changes of the atrium, and together are thought to contribute to exacerbation-related disturbances in rhythm and progression of AF [6, 10, 19–21] The understanding of COPD progression alongside AF progression is unclear [19]. Few studies have examined factors associated with hospitalization for incident AF after an exacerbation, with the focus on the short-term [21, 22]. Several have however investigated factors associated with concurrent AF at time of an exacerbation [18, 23].

Therefore, using a primary-care defined COPD cohort from the Clinical Practice Research Datalink (CPRD) Aurum database linked with hospital, mortality, and socioeconomic deprivation data, we explored factors associated with incident hospitalization for non-fatal HF, or AF or atrial flutter, within the 30 days following a COPD exacerbation.

## Study design and methods

### Data source

We obtained pseudonymized, routinely-collected electronic healthcare record data from CPRD Aurum, [24] a primary care database broadly representative by age, sex, deprivation, and regional distribution, [25] and covering approximately 20% of GP-registered patients in England [24]. Primary care data from the May 2022 build [24] were linked to the Office for National Statistics (ONS) for mortality, socioeconomic data from the 2015 Index of Multiple Deprivation (IMD), and secondary care data from NHS England’s Hospital Episode Statistics (HES) Admitted Patient Care (APC) database.

### Source population

The source population were people with a COPD diagnosis who had been included in the Exacerbations of COPD and their OutcomeS on CardioVascular diseases (EXACOS-CV) observational open cohort study [4]. People were eligible for inclusion in that original cohort study if they met the following criteria: 1) aged 40 years or older, 2) had a COPD diagnosis using a validated algorithm (86.5% PPV) [26] 3) were eligible for linkage with HES, ONS, and IMD data, 4) had a smoking history (i.e., current or ex-smoker), 5) had continuous GP practice registration with acceptable quality data in the year before start of follow up, and 6) had data recorded after 1^st^ of January 2014.

### Study design and population

**Fig. 1:**
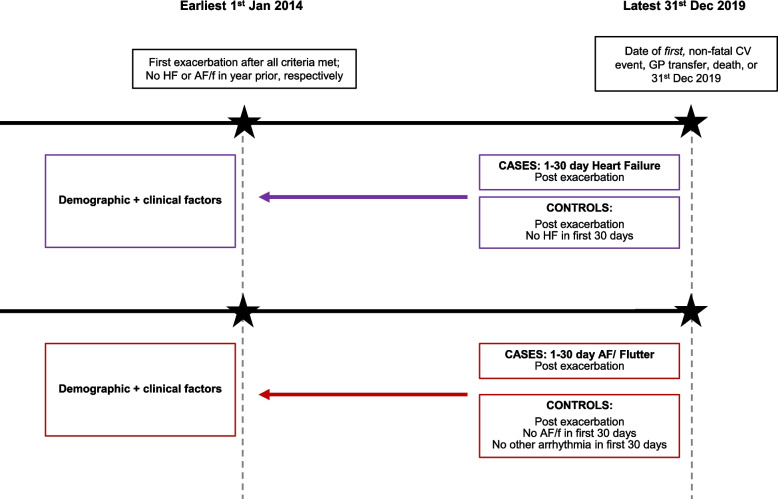
Study design for the parallel, nested case control studies. Abbreviations: HF=heart failure, AF/f = atrial fibrillation/flutter, GP=general practice, CV=cardiovascular. Two nested case control studies, for HF and AF/f respectively, were conducted among all patients who experienced an exacerbation at start of cohort follow up. Full definitions of demographic and clinical factors of interest are in the Supplementary materials

We conducted two nested-case control studies, in parallel. The study population for each study consisted of COPD patients in the EXACOS-CV source population, who 1) experienced an exacerbation within cohort follow-up, 2) among the pool of eligible controls had at least 30 days of contributing data post-exacerbation, and 3) had no evidence of HF or arrhythmias in the year before exacerbation. People were excluded from the AF/flutter study population if, within the 30 days post-exacerbation, they had evidence of other non-AF arrhythmias (e.g., cardiac arrest) (Fig. 1 for study designs; Supplementary Figures 1 and 2 for HF and AF/flutter eligibility diagram respectively).

Cases were individuals with a hospitalized, cardiovascular event (HF or AF/flutter, respectively) within 1-30 days post COPD exacerbation. Cases were determined in the HES database using ICD-10 codes in the primary diagnostic position across all episodes in a single hospitalization. The date of admission was the date of the case (index date, HF or AF/flutter, respectively). Extended case definitions are in Supplementary Table 3.

The set of controls per case was drawn among individuals in the study population who had no in-hospital diagnosis of the cardiovascular event-of-interest (HF or AF/flutter, respectively) and who had 30 days of contributing data post-exacerbation. Controls were assigned a pseudo-end date (i.e., 30 days post-exacerbation) on which to match. Controls were individually matched to cases on GP practice to account for unmeasurable potential clinical differences in disease management by clinicians, within a 30-day window of the index date, and could be used as a control for more than one case. For the HF analysis, controls were matched 2:1 and for the AF/flutter analysis, 3:1.

The choice of matching factors and ratios were chosen based on considerations of 1) maximizing the ratio itself for reasons of power and precision while 2) minimizing cases lost without a full set of controls. Only case sets with the full ratio of controls per case were included for analysis.

### Factors

We examined factors potentially associated with hospitalization for HF or AF/flutter in the 30 days following an exacerbation of COPD. Factors included demographic characteristics: age (categorized into four age bands; 40-69, 70-74, 75-79, ≥80), sex, IMD quintiles, and ethnicity; and smoking status. Comorbidities included (hypertension, anxiety, depression and depressive symptoms, type II diabetes, chronic kidney disease (CKD), BMI [body mass index; using World Health Organization classification]). COPD factors included GOLD grade of airflow limitation (defined as 1 mild, 2 moderate, 3-4 severe/very severe), Medical Research Council (MRC) dyspnea score (1-2; 3; 4-5), exacerbation severity at cohort entry (moderate/severe) prior exacerbation frequency (infrequent (≤1) versus frequent (≥2) history in a year window preceding one year to cohort entry). Prior CVD history was evidence of the following anytime preceding the year before exacerbation date [acute coronary syndrome (ACS), arrhythmias, HF, ischemic stroke, pulmonary hypertension PH], COPD inhaled therapies, and major classes of cardiovascular medications (prescriptions defined two years before cohort entry). Covariate definitions are in Supplementary Table 4.

We used validated definitions for COPD exacerbations. A moderate exacerbation was defined as a COPD-related primary care (GP) visit with either a code for exacerbation (including Lower Respiratory Tract Infection (LRTI) SNOMED-CT codes) and/or prescription for respiratory antibiotics and oral systemic corticosteroids not on the same day as an annual review, as validated in CPRD [27]. A severe exacerbation was defined as a hospitalization with an acute respiratory event code including COPD or bronchitis as a primary diagnosis, or a secondary diagnosis of COPD, as validated in HES [28]. We considered exacerbations to be the same event if recorded within 14 days in which case the highest level of severity was chosen.

We checked covariate missingness to assess use in adjusted models. Imputation was not considered for covariates with missing data, given the missingness mechanism was Missing-Not-At-Random (MNAR), violating the Missing-Completely-At-Random (MCAR) assumption premising imputation [29].

### Codelists

Codelists for primary care factors were generated using SNOMED-CT and British National Formulary ontologies; we used our standardizable, reproducible methodology, available on GitHub: for drug [30] and medical/phenotype codelists, respectively. Codelists for hospitalizations used ICD-10 codes. Codelists are available on our EXACOS-CV GitHub repository.

### Statistical analysis

We compared separately the odds of hospitalization for HF or AF/flutter between the comparator groups for each factor, using conditional logistic regression. Our final model was adjusted for all covariates without substantial amount of missing data, including demographic factors, comorbidities, and COPD inhaled therapies and cardiovascular medications. In three sensitivity analyses, we repeated main analyses additionally adjusting for variables-of-interest with substantial missing data, respectively: GOLD, MRC, and CKD.

Analyses were performed using STATA v17.

### Ethics

These data were collected and provided by CPRD. Ethical approval was obtained through CPRD’s Research Data Governance Process (protocol number: 22_002377). The RECORD checklist for observational studies is in Supplementary Table 5.

## Results

### Characteristics of study participants

The HF dataset consisted of 1,569 cases experiencing an HF event within the first 30 days post-exacerbation, matched to 3,138 controls. The AF/flutter dataset consisted of 841 cases experiencing an AF/flutter event within the first 30 days post-exacerbation, matched to 2,523 controls. Tables 1 and 2 show the characteristics of the participants for HF and AF/flutter, respectively.

**Table 1:**
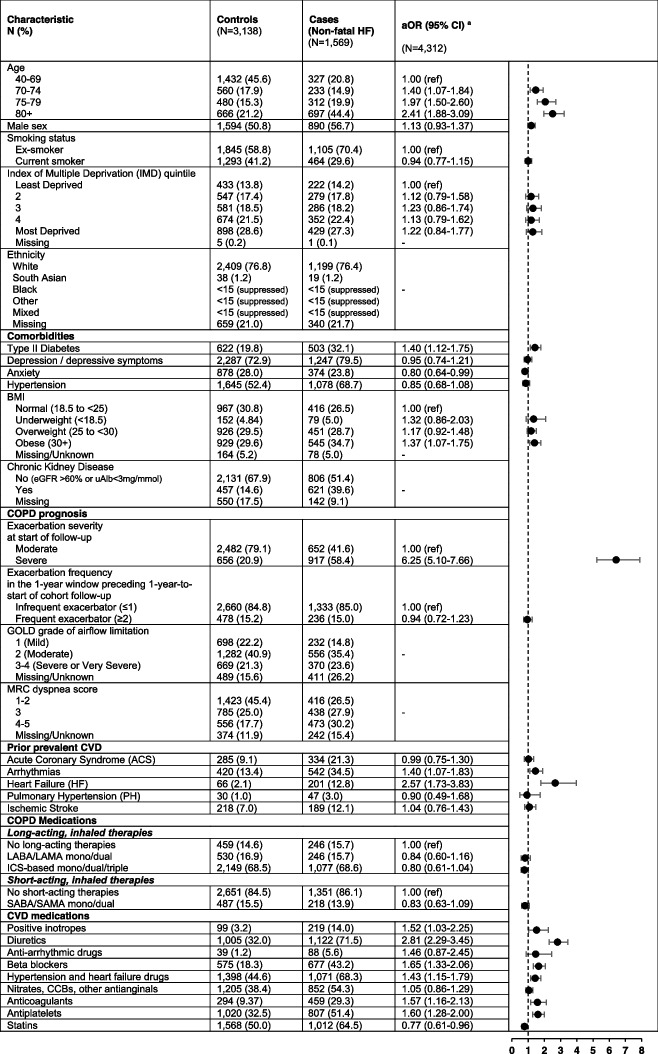
Factors associated with hospitalizations for heart failure within the first 30 days post acute exacerbation of COPD

*eGFR* Estimated glomerular filtration rate, *uAlb* Urine albumin, *CVD *Cardiovascular disease, *ICS *Inhaled corticosteroid, *LABA* Long-acting beta agonist, *LAMA *Long-acting muscarinic antagonist, *SABA *Short-acting beta agonist, *SAMA *Short-acting muscarinic antagonist, *CCB *Calcium channel blocker. n(%) described for the COPD and CV prescription categories may be mutually exclusive, as they can be taken in combination elsewhere in the table (e.g., patient prescribed a long-acting inhaler, short-acting inhaler, oral therapy, and a cardiovascular medication)

^**a**^The final model was adjusted for socioeconomic status (IMD), smoking status, and comorbidities: BMI, Type II diabetes, depression and depressive symptoms, exacerbation severity, exacerbation frequency and prior prevalent CVD (hypertension, acute coronary syndrome, arrhythmia, heart failure, pulmonary hypertension, ischemic stroke), and COPD medications (short-acting and long-acting COPD inhaled therapies) and major classes of CVD medications according to the British National Formulary (BNF)

**Table 2:**
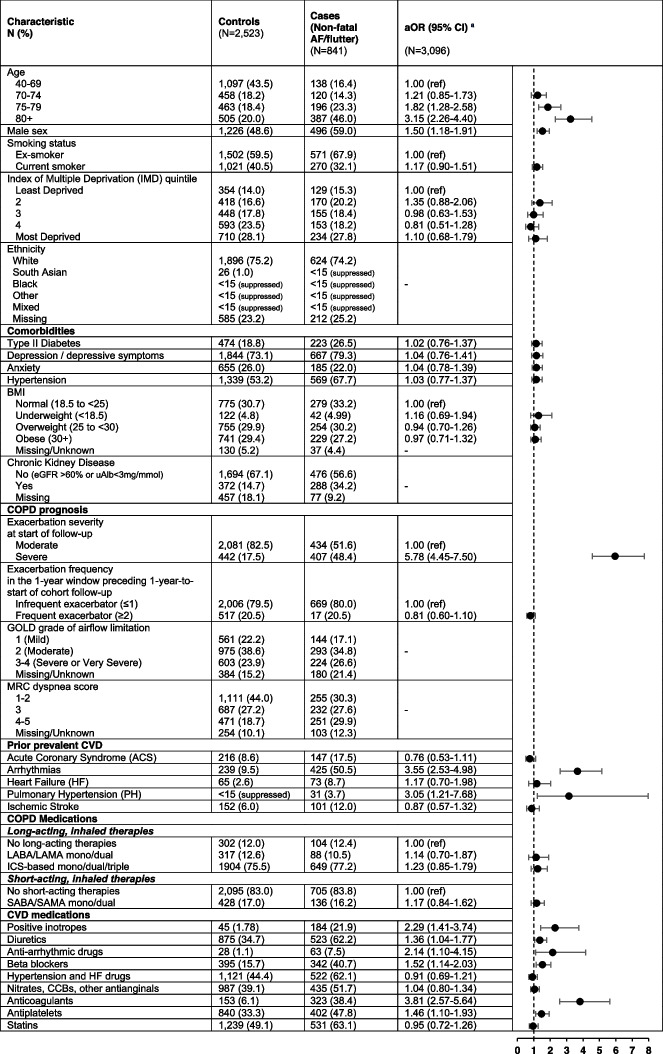
Factors associated with hospitalizations for atrial fibrillation or flutter within the first 30 days post acute exacerbation of COPD

*eGFR* Estimated glomerular filtration rate, *uAlb *Urine albumin, *CVD *Cardiovascular disease, *ICS *Inhaled corticosteroid, *LABA *Long-acting beta agonist, *LAMA* Long-acting muscarinic antagonist, *SABA *Short-acting beta agonist, *SAMA* Short-acting muscarinic antagonist, *CCB* Calcium channel blocker. n(%) described for the COPD and CV prescription categories may be mutually exclusive, as they can be taken in combination elsewhere in the table (e.g., patient prescribed a long-acting inhaler, short-acting inhaler, oral therapy, and a cardiovascular medication)

^a^The final model was adjusted for socioeconomic status (IMD), smoking status, and comorbidities: BMI, Type II diabetes, depression and depressive symptoms, exacerbation severity, exacerbation frequency and prior prevalent CVD (hypertension, acute coronary syndrome, arrhythmia, heart failure, pulmonary hypertension, ischemic stroke), and COPD medications (short-acting and long-acting COPD inhaled therapies) and major classes of CVD medications according to the British National Formulary (BNF)

Across both study populations, cases tended to have had a severe exacerbation, were more likely to be older, male, an ex-smoker, have comorbidities including prior prevalent cardiovascular disease, and be prescribed cardiovascular medications. Cases and controls both tended to have infrequent exacerbation history, have prescriptions for long-acting therapies, but tended to not have prescriptions for short-acting inhaled therapies.

### Factors associated with HF hospitalization 1-30 days post exacerbation

Age, type II diabetes, obesity, prior HF diagnosis, prior arrhythmia diagnosis, having a severe exacerbation, and most cardiovascular medications were associated with increased odds of being hospitalized for HF within 30 days of a COPD exacerbation (Table 1). The factors most strongly associated with HF were exacerbation severity (aOR=6.25, 95%CI 5.10-7.66), a prior HF diagnosis (aOR=2.57, 95%CI 1.73-3.83), age at least 80 years (≥80 vs. 40-69; aOR=2.41, 95%CI 1.88-3.09), and, of the cardiovascular medications, diuretics (aOR=2.81, 95%CI 2.29-3.45).

In sensitivity analyses, GOLD grade, MRC score, and history of CKD were all associated with an increased odds of being hospitalized for HF within the month post exacerbation.(Supplementary Table 1) The strongest associations were for CKD (aOR=1.85, 95%CI 1.46-2.35) and higher levels of airflow limitation and breathlessness (GOLD grade 3-4 Severe/Very Severe aOR=1.83, 95%CI 1.32-2.54, versus GOLD grade 1 Mild) (Score 4-5 MRC aOR=1.87, 95%CI 1.42-2.46, versus MRC 1-2).

### Factors associated with AF/flutter hospitalization 1-30 days post exacerbation

Age, male sex, prior arrhythmia, prior PH, and having a severe exacerbation were associated with AF/flutter in the 30 days following an exacerbation. Most cardiovascular medications were also associated with AF/flutter (Table 2). The factors most strongly associated with AF were exacerbation severity (aOR=5.78 95%CI 4.45-7.50), age ≥80 years (aOR=3.15 95%CI 2.26-4.40), prior arrhythmia and PH (aOR=3.55, 95%CI 2.53-4.98; aOR=3.05, 95%CI 1.21-7.68), and of the cardiovascular medications, anticoagulants (aOR=3.81, 95%CI 2.57-5.64), positive inotropes (aOR=2.29, 95%CI 1.41-3.74) and anti-arrhythmic drugs (aOR=2.14, 95%CI 1.10-4.15).

In sensitivity analyses, GOLD grade, MRC score, and CKD had no association with AF/flutter (Supplementary Table 2).

## Discussion

In a primary care defined COPD population, this study examined the clinical profiles of people hospitalized for HF and for AF within a month post exacerbation. We observed that the odds of HF and of AF hospitalization were higher for people with severe, hospitalized exacerbations and with cardiovascular-related history. For HF analyses only, the odds of HF were also higher for people with microvascular factors (i.e., type II diabetes; obesity; CKD) and for people with pulmonary factors, namely those with worse GOLD grade of airflow limitation and higher levels of MRC breathlessness scores.

### Heart failure

The pathophysiological links between COPD exacerbations and HF are recognized [31–33]. Upon exacerbation, dynamic lung hyperinflation from airflow limitation alongside heightened inflammation and hypoxia, can lead to increased strain on both the lungs and heart. The increased cardiopulmonary pressure can then lead to impaired contraction or filling of the left ventricle, namely HF with preserved or with reduced ejection fraction, respectively [31–33].

Few studies have investigated factors associated with HF in COPD alone, [15, 34–36] of which only one [15] investigated hospitalized exacerbation with concurrent HF, but did not quantify this relationship with ORs, and was conducted in the US National Inpatient Sample (NIS) database. The remaining were post-hoc analyses of trials focused on stable COPD [34–36].

The strong magnitude of the association for exacerbation severity (hospitalization) but not for exacerbation frequency, suggests two points. First, from a healthcare service-level standpoint, patients hospitalized for exacerbations are more likely to be hospitalized for a future HF (i.e., re-admission) compared with patients whose exacerbation was managed in primary care. Second, clinically, exacerbation severity (e.g., greater intensity of inflammation) has a greater indication of a patients’ future cardiac state, rather than past exacerbation occurrence and management. Findings for older age were anticipated and align with previous studies [15, 34–36].

The associations for history of HF, arrhythmia, type II diabetes, and CKD with post-exacerbation HF are unsurprising given their known independent relationships each with HF and exacerbations alone. Chronic, unmanaged HF can lead to future health service utilization for HF [16, 37]. Arrhythmia-attributed cardiac remodeling can contribute to development of cardiomyopathy [38]. Diabetes is a risk factor for substantial HF progression [16, 39], and separately a population-based study in COPD patients demonstrated an increased risk of cardiovascular mortality with type II diabetes [40]. Impaired renal hemodynamics and activation of the renin-angiotensin-aldosterone system (RAAS) can lead to HF, [41] and separately reduced kidney function is associated with future HF [42].

Our findings for cardiovascular medications indicate a certain treatment profile in primary care, leading up to the post-exacerbation HF hospitalization. The strongest association for diuretics suggests that leading up to future HF, patients perhaps are receiving treatment indicated for uncontrolled edema from existing HF, diabetic cardiomyopathy, or CKD for example.

GOLD and MRC as factors for post-exacerbation HF likewise were expected. Increased breathlessness and reduced lung function are not only symptoms of an imminent exacerbation or HF; equally, these factors can also indicate delayed diagnosis of unstable COPD or HF, [16, 43] given their shared symptomology [3, 12, 14]. Reduced lung function can contribute to worsening prognosis and precipitate a future exacerbation or HF [16, 43].

### Atrial fibrillation

The pathophysiological mechanisms implicating AF post COPD exacerbation are also established [19, 44]. At time of exacerbation, drastic increases in lung hyperinflation and impaired intrathoracic pressures can cause increased pulmonary vascular resistance and damage, leading to alterations to atrial electrophysiology [19, 44]. Compromised gas exchange in the lungs can induce systemic inflammation and oxidative stress too, and also put strain on pulmonary vasculature, leading to abnormal atrial structure and ion-channel remodeling, [19, 44] while certain treatments prescribed upon exacerbation are arrhythmogenic [19, 44].

Only four studies have investigated patient profiles for AF development in unstable COPD, all hospital-based [18, 21–23], of which two conducted in the US National Inpatient Sample (NIS) database [18, 22]. Two examined factors associated with AF diagnosis after exacerbation [21, 22] one of which patients had existing AF [21]. Two failed to quantify with ORs, only comparing baseline characteristics of exacerbating patients by status of concurrent AF [18, 23].

Our findings for older age and male gender are not unexpected; studies similarly found these associations in exacerbating [18, 22] and in stable COPD [45]. Unsurprisingly, exacerbation severity associated with future AF, again adding to the existing evidence of stronger associations for hospitalized exacerbation [1, 6] and again, likewise to HF, suggests a distinction between healthcare service-level patient pathways, and intensity versus frequency.

The associations we found for history of PH and arrhythmias aligns with what was anticipated clinically. Electrophysiological and structural changes to the atrium over time, from either AF itself [20] or from chronic atrial stretching and fibrosis attributed to PH, [46] can lead to future AF. While a study using Euro Heart Survey data showed COPD as a factor for progression of paroxysmal to persistent AF (aOR=1.51, 95%CI 0.95-2.39) [20], neither of the two studies looking at patient profiles for post-exacerbation AF, looked at chronic, prevalent arrhythmias itself as a factor [21, 22]. No studies have looked at PH, although the study among end-stage COPD patients in the NIS database found a weak association for pulmonary circulatory disorders (aOR=1.44, 95%CI 1.37-1.52) [22], compared the OR of about 3 for PH. A study found raised pulmonary artery pressure to be associated with AF (p<0.05), but failed to quantify, and it was small, underpowered, and not generalizable as it restricted to hospitalized COPD patients with existing AF [21].

The lack of associations for prior ACS, ischemic stroke, HF, and hypertension somewhat contradict the study among hospitalized, end-stage COPD patients, [22] where an association was found for HF (aOR=2.42; 95%CI: 2.36-2.48) and coagulopathy (aOR=1.23; 95%CI:1.16-1.31), but again this may reflect the more severe prognosis of these patients versus those in our study.

Likewise to HF, findings for cardiovascular medications indicate a certain treatment profile in primary care, leading up to the post-exacerbation AF hospitalization. Although we were unable to adjudicate by specific subtype of AF,(e.g., paroxysmal, persistent) the strongest associations for positive inotropes, anti-arrhythmic drugs, and anticoagulants may suggest that leading up to future AF, patients perhaps are receiving treatment to manage abnormal heart rate and/or rhythm, and/or to prevent clotting. Future research could investigate the respective treatment profile relative to paroxysmal AF and to persistent AF [47] to confirm and extend our findings.

Our null results for other comorbidities (i.e., depression and depressive symptoms, anxiety, BMI, CKD, and type-II diabetes) goes against studies finding an association for diabetes, [18, 22] mixed findings for depression, [18, 22] among other comorbidities. Yet these observed associations could be due to differing context; using the NIS database in a study population of only hospitalized, exacerbating, insured payors. GOLD airflow obstruction and MRC dyspnea score were not associated with post-exacerbation AF hospitalization, possibly as AF is often associated with vague symptoms of onset and not necessarily immediately thought about as a cause of increasing breathlessness in someone with COPD [44, 48, 49].

### Methodological considerations

A key strength is our generalizable COPD cohort, defined within the electronic healthcare record with detailed data to examine and adjust for a range of factors. Unlike other studies, this allowed us to look at two patient pathways: cardiovascular-related hospitalizations post primary-care exacerbation, and re-admissions post hospitalized exacerbation. Our exclusion criteria allowed us to quantify the odds of new onset HF and AF hospitalizations following exacerbation, by ensuring no evidence of AF or HF in the year prior to exacerbation. We chose to study two common cardiovascular conditions in COPD, AF and HF. We could not subdivide HF and AF more granularly, because of insufficient statistical power and the inability to obtain electrocardiogram or echocardiogram results to adjudicate.

We used validated codes to define COPD [26] and COPD exacerbations [27, 28] so misclassification is unlikely. Where possible, we used previously tested methods [30] and codes to define our factors-of-interest and codes were checked by a pulmonologist and/or cardiologist. The nested case control matched design allowed us to control for unmeasurable potential clinical differences in disease management by clinicians, by matching patients on GP practice.

To minimize selection bias among patients with measured factors only, we adjusted only for covariates without substantial missing data. We reserved GOLD, MRC, and CKD for sensitivity analyses; the associations of these factors with HF are generalizable only to patients with measurements (e.g., patients with greater healthcare monitoring, provision, or access). For this reason, the relationship for ethnicity could not be quantified, and given the data sparsity. For the HF analysis, we were unable to quantify B-type natriuretic peptide testing as a factor (BNP or NT-proBNP) because of 90% missing data for BNP (data not shown).

Confounding by indication is possible, particularly for the associations observed for cardiovascular medications (cases could have been more likely prescribed cardiovascular medications to manage a prevalent co-morbidity (perhaps with delayed diagnosis) that posed future cardiovascular risk, compared with controls) [50]. For example, although 12.8% of HF cases had prevalent HF diagnoses at baseline (201/1569), over 70% of HF cases were prescribed diuretics (1122/1569). Diuretics, particularly extended use of loop diuretics, can indicate possible, pre-HF diagnoses [51], given HF diagnoses tend to be delayed in COPD patients [14, 37, 51]. Taking this information together, this suggests a substantial proportion of cases could have been prescribed diuretics to manage possible-yet-undiagnosed HF, in which case, the later case-defining hospitalization was the delayed, first-time diagnosis of HF.

Our results for cardiovascular medications do not imply these medications are increasing the cardiovascular risk, rather they add to an understanding of the exacerbating patient profile. Furthermore, although these medications could indicate delayed CVD diagnosis, alternatively they could be medically indicated for management of a co-morbidity we did not adjust for, e.g., beta-blockers can be prescribed for thyroid conditions [52].

### Implications for clinical practice

Within the month-window following an exacerbation, largely exacerbation intensity and cardiovascular-related management and disease history were associated with odds of incident HF and AF. For HF specifically, existing type II diabetes, CKD, lung function (GOLD grade), and levels of breathlessness (MRC) had an association too—but not for AF. These factors can help better identify patients most at-risk for HF and AF, to streamline efforts to allocate screening, vigilant monitoring, and prevention.

At the time of a COPD exacerbation, particularly hospitalized exacerbation, we recommend preemptively monitoring markers of possible HF, through taking medication history of loop diuretics, [51] and through BNP testing [53]. At present however, HF prevention is narrow in scope, with guidelines for early identification of HF not explicitly considering unstable COPD [53, 54]. Our results suggest that HF monitoring should widen to include patients with COPD exacerbations.

Upon exacerbation, particularly hospitalized exacerbation, we recommend proactively screening for AF (e.g., electrocardiogram) [19] as AF commonly presents subclinically [17]. Still, at present, AF screening is narrow in scope; it is primarily conducted in patients with existing or suspected AF with the goal of preventing stroke, with AF guidelines not explicitly considering unstable COPD [17, 55]. Our results suggest that AF screening should widen to include patients with COPD exacerbations, to help prevent future AF—even before stroke.

## Conclusions

Cardiopulmonary factors were associated with hospitalization for HF in the 30 days following a COPD exacerbation, while only cardiovascular-related factors and exacerbation severity were associated with AF hospitalization. Understanding risk factors will help to target people for prevention. Future studies with the intent of improving risk prediction for HF and AF should consider exacerbations of COPD.

### Supplementary Information

**Supplementary Material 1.** 

## Data Availability

Data are available on request from the CPRD. Their provision requires the purchase of a license, and this license does not permit the authors to make them publicly available to all. This work used data from the CPRD Aurum version collected in May 2022 and have clearly specified the data selected within the Methods section, and linked data in the Supplementary Materials. To allow identical data to be obtained by others, via the purchase of a license, the code lists will be provided upon request. Licenses are available from the CPRD (http://www.cprd.com): The Clinical Practice Research Datalink Group, The Medicines and Healthcare products Regulatory Agency, 10 South Colonnade, Canary Wharf, London E14 4PU.

## Abbreviations

COPD: Chronic Obstructive Pulmonary Disease
CPRD: Clinical Practice Research Datalink
CVD: Cardiovascular Disease
GP: General practice
HES: Hospital Episode Statistics
IMD: Index of Multiple Deprivation
MRC: Medical Research Council
HF: Heart Failure
AF: Atrial Fibrillation
CKD: Chronic Kidney Disease

## References

[1] Müllerová H, Marshall J, de Nigris E, Varghese P, Pooley N, Embleton N, et al. Association of COPD exacerbations and acute cardiovascular events: a systematic review and meta-analysis. Ther Adv Respir Dis [Internet]. 2022 Jan;16:175346662211136. Available from: 10.1177/17534666221113647. 10.1177/17534666221113647 PMC9340406 35894441

[2] Morgan AD, Zakeri R, Quint JK. Defining the relationship between COPD and CVD: what are the implications for clinical practice? Ther Adv Respir Dis [Internet]. 2018 Jan 1;12:175346581775052. Available from: 10.1177/1753465817750524. 10.1177/1753465817750524 PMC5937157 29355081

[3] Balbirsingh V, Mohammed AS, Turner AM, Newnham M. Cardiovascular disease in chronic obstructive pulmonary disease: a narrative review. Thorax [Internet]. 2022 Sep;77(9):939–45. Available from: https://thorax.bmj.com/lookup/doi/10.1136/thoraxjnl-2021-218333. 10.1136/thoraxjnl-2021-218333 35772939

[4] Graul EL, Nordon C, Rhodes K, Marshall J, Menon S, Kallis C, et al. Temporal risk of non-fatal cardiovascular events post COPD exacerbation: a population-based study [Accepted 20 December 2023]. Am J Respir Crit Care Med [Internet]. 2023 Dec; Available from: 10.1164/rccm.202307-1122OC. 10.1164/rccm.202307-1122OC PMC11531205 38127850

[5] Rothnie KJ, Connell O, Müllerová H, Smeeth L, Pearce N, Douglas I, et al. Myocardial Infarction and Ischemic Stroke after Exacerbations of Chronic Obstructive Pulmonary Disease. Ann Am Thorac Soc [Internet]. 2018 Aug;15(8):935–46. Available from: 10.1513/AnnalsATS.201710-815OC. 10.1513/AnnalsATS.201710-815OC PMC6322039 29723057

[6] Hirayama A, Goto T, Shimada YJ, Faridi MK, Camargo CA, Hasegawa K. Acute Exacerbation of Chronic Obstructive Pulmonary Disease and Subsequent Risk of Emergency Department Visits and Hospitalizations for Atrial Fibrillation. Circ Arrhythm Electrophysiol [Internet] (2018). Sep; 11(9):e006322. Available from:.

[7] Halpin DMG, Decramer M, Celli B, Kesten S, Leimer I, Tashkin DP. Risk of Nonlower Respiratory Serious Adverse Events Following COPD Exacerbations in the 4-year UPLIFT® Trial. Lung [Internet]. 2011 Aug [cited 2022 Oct 11];189(4):261–8. Available from: 10.1007/s00408-011-9301-8. 10.1007/s00408-011-9301-8 PMC3140935 21678045

[8] Goto T, Shimada YJ, Faridi MK, Camargo CA, Hasegawa K. Incidence of Acute Cardiovascular Event After Acute Exacerbation of COPD. J Gen Intern Med [Internet]. 2018 Sep;33(9):1461–8. Available from: 10.1007/s11606-018-4518-3. 10.1007/s11606-018-4518-3 PMC6108996 29948806

[9] Swart KMA, Baak BN, Lemmens L, Penning-van Beest FJA, Bengtsson C, Lobier M, Risk of cardiovascular events after an exacerbation of chronic obstructive pulmonary disease: results from the EXACOS-CV cohort study using the PHARMO Data Network in the Netherlands. Respir Res [Internet]., et al. 21;24(1):293. Available from: 2023 Nov. 10.1186/s12931-023-02601-4. 10.1186/s12931-023-02601-4 PMC10662240 37990197

[10] Chen W, Thomas J, Sadatsafavi M, FitzGerald JM. Risk of cardiovascular comorbidity in patients with chronic obstructive pulmonary disease: a systematic review and meta-analysis. Lancet Respir Med [Internet]. 2015 Aug;3(8):631–9. Available from: 10.1016/S2213-2600(15)00241-6. 10.1016/S2213-2600(15)00241-6 26208998

[11] Hesse K, Bourke S, Steer J. Heart failure in patients with COPD exacerbations: Looking below the tip of the iceberg. Respir Med [Internet] (2022). May; 196:106800. Available from:.

[12] Cuthbert JJ, Pellicori P, Optimal Clark AL., Management of Heart Failure and Chronic Obstructive Pulmonary Disease: Clinical Challenges. Int J Gen Med [Internet]. [cited 2023 Aug 9];Volume 15:7961–75. Available from: 2022Oct. 10.2147/IJGM.S295467. 10.2147/IJGM.S295467 PMC9617562 36317097

[13] MacDonald DM, Ji Y, Adabag S, Alonso A, Chen LY, Henkle BE, et al. Cardiovascular Autonomic Function and Incident COPD Hospitalizations in ARIC. Ann Am Thorac Soc [Internet]. 2023 Jun 26;AnnalsATS.202211-964OC. Available from: 10.1513/annalsats.202211-964oc. 10.1513/AnnalsATS.202211-964OC PMC10559138 37364277

[14] Hayhoe B, Kim D, Aylin PP, Majeed FA, Cowie MR, Bottle A. Adherence to guidelines in management of symptoms suggestive of heart failure in primary care. Heart [Internet]. 2019 May;105(9):678–85. Available from: 10.1136/heartjnl-2018-313971. 10.1136/heartjnl-2018-313971 30514731

[15] Kichloo A, Minhas AMK, Jamal S, Shaikh AT, Albosta M, Singh J, Trends and Inpatient Outcomes of Primary Heart Failure Hospitalizations with a Concurrent Diagnosis of Acute Exacerbation of Chronic Obstructive Pulmonary Disease (from The National Inpatient Sample Database from 2004 to 2014). Am J Cardiol [Internet]., et al. Jul; 150:69–76. Available from: 2021. 10.1016/j.amjcard.2021.03.054. 10.1016/j.amjcard.2021.03.054 34001343

[16] Axson EL, Ragutheeswaran K, Sundaram V, Bloom CI, Bottle A, Cowie MR, Hospitalisation and mortality in patients with comorbid COPD and heart failure: a systematic review and meta-analysis. Respir Res [Internet]., et al. [cited 2023 Apr 27];21(1):54. Available from: 2020Dec. 10.1186/s12931-020-1312-7. 10.1186/s12931-020-1312-7 PMC7023777 32059680

[17] US Preventive Services Task Force, Davidson KW, Barry MJ, Mangione CM, Cabana M, Caughey AB, et al. Screening for Atrial Fibrillation: US Preventive Services Task Force Recommendation Statement. JAMA [Internet]. 2022 Jan 25;327(4):360. Available from: 10.1001/jama.2021.23732. 10.1001/jama.2021.23732 35076659

[18] Abdullah AS, Eigbire G, Ali M, Mohanad A, Abdul W, Hisham I, et al. Relationship of Atrial Fibrillation to Outcomes in Patients Hospitalized for Chronic Obstructive Pulmonary Disease Exacerbation. J Atr Fibrillation [Internet]. 2019 Aug [cited 2023 Aug 7];12(2):2117. Available from: 10.4022/jafib.2117. . 10.4022/jafib.2117 PMC6990049 32002109

[19] Simons SO, Elliott A, Sastry M, Hendriks JM, Arzt M, Rienstra M, et al. Chronic obstructive pulmonary disease and atrial fibrillation: an interdisciplinary perspective. Eur Heart J [Internet]. 2021 Feb 1 [cited 2023 Aug 11];42(5):532–40. Available from: 10.1093/eurheartj/ehaa822. 10.1093/eurheartj/ehaa822 33206945

[20] De Vos CB, Pisters R, Nieuwlaat R, Prins MH, Tieleman RG, Coelen RJS, et al. Progression From Paroxysmal to Persistent Atrial Fibrillation. J Am Coll Cardiol [Internet]. 2010 Feb;55(8):725–31. Available from: 10.1016/j.jacc.2009.11.040. 10.1016/j.jacc.2009.11.040 20170808

[21] Terzano C, Romani S, Conti V, Paone G, Oriolo F, Vitarelli A. Atrial fibrillation in the acute, hypercapnic exacerbations of COPD. Eur Rev Med Pharmacol Sci [Internet]. 2014 Oct;18(19):2908–17. Available from: https://www.europeanreview.org/article/7899. 25339486

[22] Xiao X, Han H, Wu C, He Q, Ruan Y, Zhai Y, et al. Prevalence of Atrial Fibrillation in Hospital Encounters With End-Stage COPD on Home Oxygen. Chest [Internet]. 2019 May [cited 2023 Aug 7];155(5):918–27. Available from: 10.1016/j.chest.2018.12.021. 10.1016/j.chest.2018.12.021 30684473

[23] Rusinowicz T, Zielonka TM, Zycinska K. Cardiac Arrhythmias in Patients with Exacerbation of COPD. In: Clinical Management of Pulmonary Disorders and Diseases [Internet]. Cham: Springer International Publishing; 2017 [cited 2023 Aug 7]. p. 53–62. (Advances in Experimental Medicine and Biology; vol. 1022). Available from: http://link.springer.com/10.1007/5584_2017_41. 10.1007/5584_2017_41 28573445

[24] Clinical Practice Research Datalink. CPRD Aurum May 2022 (Version 2022.05.001) [Internet]. Clinical Practice Research Datalink; 2022 [cited 2022 Nov 30]. Available from: 10.48329/t89s-kf12.

[25] Wolf A, Dedman D, Campbell J, Booth H, Lunn D, Chapman J, et al. Data resource profile: Clinical Practice Research Datalink (CPRD) Aurum. Int J Epidemiol [Internet]. 2019 Dec 1;48(6):1740–1740g. Available from: 10.1093/ije/dyz034. 10.1093/ije/dyz034 PMC6929522 30859197

[26] Quint JK, Müllerova H, DiSantostefano RL, Forbes H, Eaton S, Hurst JR, et al. Validation of chronic obstructive pulmonary disease recording in the Clinical Practice Research Datalink (CPRD-GOLD). BMJ Open [Internet]. 2014 Jul 1;4(7):e005540. Available from: 10.1136/bmjopen-2014-005540. 10.1136/bmjopen-2014-005540 PMC4120321 25056980

[27] Rothnie KJ, Müllerová H, Hurst JR, Smeeth L, Davis K, Thomas SL, et al. Validation of the Recording of Acute Exacerbations of COPD in UK Primary Care Electronic Healthcare Records. Chotirmall SH, editor. PLOS ONE [Internet]. 2016 Mar 9;11(3):e0151357. Available from: 10.1371/journal.pone.0151357. 10.1371/journal.pone.0151357 PMC4784784 26959820

[28] Rothnie KJ, Müllerová H, Thomas SL, Chandan JS, Smeeth L, Hurst JR, Recording of hospitalizations for acute exacerbations of COPD in UK electronic health care records. ClinEpidemiol [Internet]., et al. Nov;Volume 8:771–82. Available from: 2016. 10.2147/CLEP.S117867. 10.2147/CLEP.S117867 PMC5123723 27920578

[29] Carpenter JR, Smuk M. Missing data: A statistical framework for practice. Biom J [Internet]. 2021 Jun [cited 2023 Feb 28];63(5):915–47. Available from: 10.1002/bimj.202000196. 10.1002/bimj.202000196 PMC7615108 33624862

[30] Graul EL, Stone PW, Massen GM, Hatam S, Adamson A, Denaxas S, et al. Determining prescriptions in electronic healthcare record data: methods for development of standardized, reproducible drug codelists. JAMIA Open [Internet]. 2023 Jul 4 [cited 2023 Sep 1];6(3):ooad078. Available from: https://academic.oup.com/jamiaopen/article/doi/10.1093/jamiaopen/ooad078/7252957. 10.1093/jamiaopen/ooad078 PMC10463548 37649988

[31] Axson EL, Sundaram V, Bloom CI, Bottle A, Cowie MR, Quint JK (2020). Temporal Trends in the Incidence of Heart Failure among Patients with Chronic Obstructive Pulmonary Disease and Its Association with Mortality. Ann Am Thorac Soc..

[32] de Miguel Díez J, Chancafe Morgan J, Jiménez García R (2013). The association between COPD and heart failure risk: a review. Int J Chron Obstruct Pulmon Dis..

[33] Stringer WW (2020). Are We Treating Heart Failure in Patients with Chronic Obstructive Pulmonary Disease Appropriately?. Ann Am Thorac Soc..

[34] Ehteshami-Afshar S, Mooney L, Dewan P, Desai AS, Lang NN, Lefkowitz MP, Characteristics Clinical, Outcomes of Patients With Heart Failure With Reduced Ejection Fraction and Chronic Obstructive Pulmonary Disease: Insights From PARADIGM-HF. J Am Heart Assoc [Internet]., et al. 16;10(4):e019238. Available from: 2021Feb. 10.1161/jaha.120.019238. 10.1161/JAHA.120.019238 PMC7955331 33522249

[35] Mentz RJ, Schmidt PH, Kwasny MJ, Ambrosy AP, O’Connor CM, Konstam MA, et al. The Impact of Chronic Obstructive Pulmonary Disease in Patients Hospitalized for Worsening Heart Failure With Reduced Ejection Fraction: An Analysis of the EVEREST Trial. J Card Fail [Internet]. 2012 Jul [cited 2023 Aug 15];18(7):515–23. Available from: 10.1016/j.cardfail.2012.04.010. 10.1016/j.cardfail.2012.04.010 22748484

[36] Mentz RJ, Schulte PJ, Fleg JL, Fiuzat M, Kraus WE, Piña IL, et al. Clinical characteristics, response to exercise training, and outcomes in patients with heart failure and chronic obstructive pulmonary disease: Findings from Heart Failure and A Controlled Trial Investigating Outcomes of Exercise TraiNing (HF-ACTION). Am Heart J [Internet]. 2013 Feb;165(2):193–9. Available from: 10.1016/j.ahj.2012.10.029. 10.1016/j.ahj.2012.10.029 PMC3762597 23351822

[37] Taylor CJ. Diagnosing heart failure: challenges in primary care. Heart [Internet]. 2019 May;105(9):663–4. Available from: 10.1136/heartjnl-2018-314396. 10.1136/heartjnl-2018-314396 30647095

[38] Santhanakrishnan R, Wang N, Larson MG, Magnani JW, McManus DD, Lubitz SA, et al. Atrial Fibrillation Begets Heart Failure and Vice Versa: Temporal Associations and Differences in Preserved Versus Reduced Ejection Fraction. Circulation [Internet]. 2016 Feb 2;133(5):484–92. Available from: 10.1161/CIRCULATIONAHA.115.018614. 10.1161/CIRCULATIONAHA.115.018614 PMC4738087 26746177

[39] Echouffo-Tcheugui JB, Ndumele CE, Zhang S, Florido R, Matsushita K, Coresh J, et al. Diabetes and Progression of Heart Failure. J Am Coll Cardiol [Internet]. 2022 Jun;79(23):2285–93. Available from: 10.1016/j.jacc.2022.03.378. 10.1016/j.jacc.2022.03.378 PMC10125541 35680178

[40] Raslan AS, Quint JK, Cook S. All-Cause, Cardiovascular and Respiratory Mortality in People with Type 2 Diabetes and Chronic Obstructive Pulmonary Disease (COPD) in England: A Cohort Study Using the Clinical Practice Research Datalink (CPRD). Int J Chron Obstruct Pulmon Dis [Internet]. 2023 Jun [cited 2023 Aug 15];Volume 18:1207–18. Available from: https://www.dovepress.com/all-cause-cardiovascular-and-respiratory-mortality-in-people-with-type-peer-reviewed-fulltext-article-COPD. 10.2147/COPD.S407085 PMC10276568 37332839

[41] Ryan DK, Banerjee D, Jouhra F. Management of Heart Failure in Patients with Chronic Kidney Disease. Eur Cardiol Rev [Internet]. 2022 Jul 26;17:e17. Available from: 10.15420/ecr.2021.33. 10.15420/ecr.2021.33 PMC9376857 35990402

[42] Kottgen A, Russell SD, Loehr LR, Crainiceanu CM, Rosamond WD, Chang PP, et al. Reduced Kidney Function as a Risk Factor for Incident Heart Failure: The Atherosclerosis Risk in Communities (ARIC) Study. J Am Soc Nephrol [Internet]. 2007 Apr;18(4):1307–15. Available from: 10.1681/asn.2006101159. 10.1681/ASN.2006101159 17344421

[43] Axson EL, Bottle A, Cowie MR, Quint JK. Relationship between heart failure and the risk of acute exacerbation of COPD. Thorax. 2021 Apr 29;thoraxjnl-2020-216390. 10.1136/thoraxjnl-2020-216390 PMC8311079 33927022

[44] Ioannides AE, Tayal U, Quint JK (2023). Spirometry in atrial fibrillation: what’s the catch?. Expert Rev Respir Med..

[45] Huang Q, Xiong H, Shuai T, Zhang M, Zhang C, Wang Y, et al. Risk factors for new-onset atrial fibrillation in patients with chronic obstructive pulmonary disease: a systematic review and meta-analysis. PeerJ [Internet]. 2020 Dec 2;8:e10376. Available from: 10.7717/peerj.10376. 10.7717/peerj.10376 PMC7718784 33344074

[46] Vahdatpour CA, Luebbert JJ, Palevsky HI. Atrial arrhythmias in chronic lung disease‐associated pulmonary hypertension. Pulm Circ [Internet]. 2020 Jan;10(1):1–13. Available from: 10.1177/2045894020910685. 10.1177/2045894020910685 PMC7065292 32215200

[47] Camm AJ, Naccarelli GV, Mittal S, Crijns HJGM, Hohnloser SH, Ma CS, et al. The Increasing Role of Rhythm Control in Patients With Atrial Fibrillation. J Am Coll Cardiol [Internet]. 2022 May [cited 2024 Apr 12];79(19):1932–48. Available from: https://linkinghub.elsevier.com/retrieve/pii/S0735109722044886. 10.1016/j.jacc.2022.03.337 35550691

[48] van der Velden RMJ, Hermans ANL, Pluymaekers NAHA, Gawalko M, Elliott A, Hendriks JM (2022). Dyspnea in patients with atrial fibrillation: Mechanisms, assessment and an interdisciplinary and integrated care approach. Int J Cardiol Heart Vasc..

[49] Guerra F, Brambatti M, Nieuwlaat R, Marcucci M, Dudink E, Crijns HJGM (2017). Symptomatic atrial fibrillation and risk of cardiovascular events: data from the Euro Heart Survey. Eur Eur Pacing Arrhythm Card Electrophysiol J Work Groups Card Pacing Arrhythm Card Cell Electrophysiol Eur Soc Cardiol..

[50] Kyriacou DN, Lewis RJ. Confounding by Indication in Clinical Research. JAMA [Internet] (2016). 1;316(17):1818. Available from:.

[51] Axson EL, Bottle A, Cowie MR, Quint JK. Relationship between heart failure and the risk of acute exacerbation of COPD. Thorax [Internet]. 2021 Aug;76(8):807–14. Available from: https://thorax.bmj.com/lookup/doi/10.1136/thoraxjnl-2020-216390. 10.1136/thoraxjnl-2020-216390 PMC8311079 33927022

[52] National Institute for Health and Care Excellence. British National Formulary. 2023. Beta-adrenoceptor blocking drugs: treatment summaries. Available from: https://bnf.nice.org.uk/treatment-summaries/beta-adrenoceptor-blocking-drugs/.

[53] Heidenreich PA, Bozkurt B, Aguilar D, Allen LA, Byun JJ, Colvin MM, et al. 2022 AHA/ACC/HFSA Guideline for the Management of Heart Failure: A Report of the American College of Cardiology/American Heart Association Joint Committee on Clinical Practice Guidelines. Circulation [Internet]. 2022 May 3;145(18). Available from: 10.1161/CIR.0000000000001063. 10.1161/CIR.0000000000001063 35363499

[54] National Institute for Health and Care Excellence. Chronic heart failure in adults: diagnosis and management [NG106] [Internet]. 2018. Available from: https://www.nice.org.uk/guidance/ng106/resources/chronic-heart-failure-in-adults-diagnosis-and-management-pdf-66141541311685. 30645061

[55] National Institute for Health and Care Excellence. Atrial fibrillation: diagnosis and management [NG196] [Internet]. 2021. Available from: https://www.nice.org.uk/guidance/ng196/resources/atrial-fibrillation-diagnosis-and-management-pdf-66142085507269. 34165935

