# Supplementary material for: Factors associated with non-fatal heart failure and atrial fibrillation or flutter within the first 30 days post COPD exacerbation: a nested case-control study
Source: BMC Pulm Med. 2024 May 4;24:221. doi: 10.1186/s12890-024-03035-4 (PMC11069200; doi:10.1186/s12890-024-03035-4)
Supplement: Supplementary file 1 — Supplementary Material 1. [file 12890_2024_3035_MOESM1_ESM.docx]

**Supplementary Materials**

**Paper:** Factors associated with non-fatal heart failure and atrial fibrillation or flutter within the first 30 days post exacerbation of COPD: a nested case-control study

**Authors:** Emily L. Graul MSc, Clementine Nordon PhD, Kirsty Rhodes PhD, Shruti Menon PhD, Mahmoud Al Ammouri MD, Constantinos Kallis PhD, Anne E. Ioannides PhD, Hannah R. Whittaker PhD, Nicholas S. Peters FRCP, Jennifer K. Quint FRCP

**Date:** 13 December 2023

Table of Contents

[Appendix 1. Supplementary Figures and Tables for Heart Failure 2](#_Toc153889930)

[Figure 1. Inclusion of patients in the case control study for heart failure 2](#_Toc153889931)

[Table 1. Factors associated with hospitalizations for heart failure within the first 30 days post acute exacerbation of COPD (all models) 3](#_Toc153889932)

[Appendix 2. Supplementary Figures and Tables for Atrial Fibrillation 6](#_Toc153889933)

[Figure 2. Inclusion of patients in the case control study for atrial fibrillation/flutter 6](#_Toc153889934)

[Table 2. Factors associated with hospitalizations for atrial fibrillation or flutter within the first 30 days post acute exacerbation of COPD (minimally-adjusted models and sensitivity analyses) 7](#_Toc153889935)

[Appendix 3. Extended Methods 10](#_Toc153889936)

[Table 3. Extended case definitions 10](#_Toc153889937)

[Table 4. Extended covariate definitions 10](#_Toc153889938)

[Power calculations 12](#_Toc153889939)

[STROBE and RECORD Checklist 13](#_Toc153889940)

[Table 5. STROBE and RECORD Checklist(9) 13](#_Toc153889941)

[References for Supplementary Materials 17](#_Toc153889942)

[References for Datasets (CPRD and Linked) 18](#_Toc153889943)

# Appendix 1. Supplementary Figures and Tables for Heart Failure

## Figure 1. Inclusion of patients in the case control study for heart failure


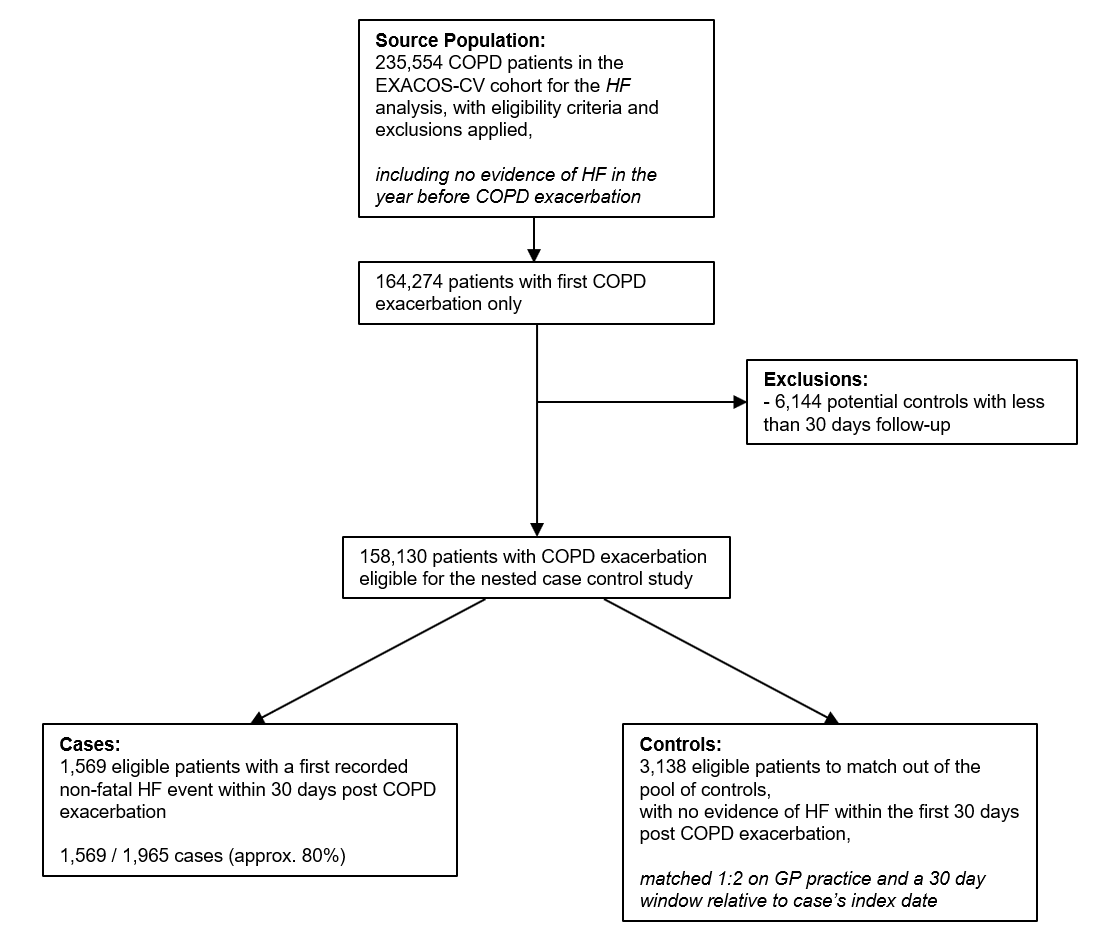

Legend: COPD (Chronic Obstructive Pulmonary Disease); GP (General Practitioner)

## Table 1. Factors associated with hospitalizations for heart failure within the first 30 days post acute exacerbation of COPD (all models)

| **Characteristic**  **N (%)** | **Minimally-adjusted**  **OR (95% CI)**  *Adjusted for age and sex*  (N=4,707 unless specified) | **Final Model**  **aOR (95% CI)**  (N=4,312) | **Sensitivity Analysis:**  **aOR (95% CI)**  *Final Model*  *+GOLD stage*  (N=2,946) | **Sensitivity Analysis**  **aOR (95% CI)**  *Final Model*  *+MRC dyspoenea score*  (N=3,410) | **Sensitivity Analysis**  **aOR (95% CI)**  *Final Model*  *+CKD*  (N=3,490) |
| --- | --- | --- | --- | --- | --- |
| Age |  |  |  |  |  |
| 40-69 | 1.00 (ref) | 1.00 (ref) | 1.00 (ref) | 1.00 (ref) | 1.00 (ref) |
| 70-74 | 1.84 (1.51-2.24) * | 1.40 (1.07-1.84) *p=0.015 | 1.46 (1.05-2.02) *p=0.024 | 1.42 (1.04-1.94) *p=0.026 | 1.34 (0.98-1.82) p=0.066 |
| 75-79 | 2.97 (2.44-3.62) * | 1.97 (1.50-2.60) * | 1.83 (1.31-2.55) * | 2.04 (1.49-2.80) * | 1.94 (1.41-2.66) * |
| 80+ | 4.70 (3.97-5.56) * | 2.41 (1.88-3.09) * | 2.47 (1.80-3.37) * | 2.47 (1.85-3.30) * | 2.03 (1.51-2.71) * |
| Male sex | 1.27 (1.11-1.44) *p=0.001 | 1.13 (0.93-1.37) p=0.21 | 1.11 (0.88-1.41) p=0.37 | 1.12 (0.90-1.39) p=0.33 | 1.08 (0.87-1.34) p=0.48 |
| Smoking status |  |  |  |  |  |
| Ex-smoker | 1.00 (ref) | 1.00 (ref) | 1.00 (ref) | 1.00 (ref) | 1.00 (ref) |
| Current smoker | 0.83 (0.72-0.96) *p=0.013 | 0.94 (0.77-1.15) p=0.54 | 0.88 (0.69-1.13) p=0.33 | 0.99 (0.79-1.25) p=0.96 | 1.01 (0.80-1.28) p=0.93 |
| Index of Multiple Deprivation (IMD) quintile |  |  |  |  |  |
| Least Deprived | 1.00 (ref) | 1.00 (ref) | 1.00 (ref) | 1.00 (ref) | 1.00 (ref) |
| 2 | 1.06 (0.83-1.36) p=0.65 | 1.12 (0.79-1.58) p=0.52 | 1.05 (0.69-1.61) p=0.81 | 1.14 (0.77-1.70) p=0.50 | 1.08 (0.73-1.60) p=0.68 |
| 3 | 1.11 (0.86-1.43) p=0.41 | 1.23 (0.86-1.74) p=0.26 | 1.27 (0.83-1.93) p=0.27 | 1.25 (0.84-1.87) p=0.27 | 1.44 (0.97-2.14) p=0.074 |
| 4 | 1.16 (0.89-1.50) p=0.27 | 1.13 (0.79-1.62) p=0.50 | 1.13 (0.74-1.74) p=0.58 | 1.14 (0.76-1.71) p=0.52 | 1.17 (0.78-1.75) p=0.44 |
| Most Deprived | 1.22 (0.93-1.60) p=0.16 | 1.22 (0.84-1.77) p=0.29 | 1.05 (0.67-1.65) p=0.83 | 1.06 (0.69-1.62) p=0.80 | 1.28 (0.84-1.95) p=0.26 |
| Missing | - | - | - | - |  |
| Ethnicity |  |  |  |  |  |
| White |  |  |  |  |  |
| South Asian |  |  |  |  |  |
| Black | - | - | - | - | - |
| Other |  |  |  |  |  |
| Mixed |  |  |  |  |  |
| Missing |  |  |  |  |  |
| **Comorbidities** |  |  |  |  |  |
| Type II Diabetes | 1.88 (1.61-2.18) * | 1.40 (1.12-1.75) *p=0.003 | 1.16 (0.89-1.52) p=0.27 | 1.33 (1.03-1.71) *p=0.028 | 1.33 (1.04-1.70) *p=0.025 |
| Depression / depressive symptoms | 1.43 (1.22-1.68) * | 0.95 (0.74-1.21) p=0.66 | 1.08 (0.80-1.44) p=0.62 | 1.05 (0.79-1.38) p=0.75 | 0.85 (0.64-1.14) p=0.28 |
| Anxiety | 1.00 (0.86-1.17) p=0.99 | 0.80 (0.64-0.99) *p=0.043 | 0.84 (0.63-1.11) p=0.22 | 0.79 (0.61-1.02) p=0.066 | 0.79 (0.62-1.01) p=0.060 |
| Hypertension | 1.55 (1.35-1.78) * | 0.85 (0.68-1.08) p=0.18 | 0.88 (0.66-1.18) p=0.40 | 0.92 (0.70-1.20) p=0.54 | 0.79 (0.61-1.03) p=0.083 |
| BMI |  |  |  |  |  |
| Normal (18.5 to <25) | 1.00 (ref) | 1.00 (ref) | 1.00 (ref) | 1.00 (ref) | 1.00 (ref) |
| Underweight (<18.5) | 1.35 (0.97-1.87) p=0.073 | 1.32 (0.86-2.03) p=0.21 | 1.35 (0.78-2.33) p=0.29 | 1.35 (0.82-2.23) p=0.24 | 1.49 (0.92-2.44) p=0.11 |
| Overweight (25 to <30) | 1.15 (0.97-1.38) p=0.11 | 1.17 (0.92-1.48) p=0.20 | 1.14 (0.86-1.52) p=0.36 | 1.10 (0.84-1.43) p=0.50 | 1.28 (0.98-1.68) p=0.073 |
| Obese (30+) | 1.67 (1.40-1.99) * | 1.37 (1.07-1.75) *p=0.012 | 1.44 (1.06-1.94) *p=0.18 | 1.31 (0.99-1.73) p=0.060 | 1.45 (1.09-1.92) *p=0.010 |
| Missing/Unknown | - | - | - | - | - |
| Chronic Kidney Disease |  |  |  |  |  |
| No (eGFR >60% or uAlb<3mg/mmol) | 1.00 (ref) |  |  |  | 1.00 (ref) |
| Yes | 2.62 (2.21-3.12) * | - | - | - | 1.85 (1.46-2.35) * |
| Missing | - |  |  |  | - |
| **COPD prognosis** |  |  |  |  |  |
| Exacerbation severity  at start of follow-up |  |  |  |  |  |
| Moderate | 1.00 (ref) | 1.00 (ref) | 1.00 (ref) | 1.00 (ref) | 1.00 (ref) |
| Severe | 5.18 (4.43-6.06) * | 6.25 (5.10-7.66) * | 6.26 (4.87-8.05) * | 6.08 (4.81-7.68) * | 6.73 (5.32-8.52) * |
| Exacerbation frequency  in the 1-year window preceding 1-year-to-start of cohort follow-up |  |  |  |  |  |
| Infrequent exacerbator (≤1) | 1.00 (ref) | 1.00 (ref) | 1.00 (ref) | 1.00 (ref) | 1.00 (ref) |
| Frequent exacerbator (≥2) | 1.06 (0.87-1.29) p=0.57 | 0.94 (0.72-1.23) p=0.66 | 0.81 (0.58-1.15) p=0.24 | 0.88 (0.64-1.19) p=0.41 | 0.91 (0.67-1.24) p=0.55 |
| GOLD grade of airflow limitation |  |  |  |  |  |
| 1 (Mild) | 1.00 (ref) |  | 1.00 (ref) |  |  |
| 2 (Moderate) | 1.45 (1.18-1.79) | - | 1.45 (1.09-1.93) *p=0.010 | - | - |
| 3-4 (Severe or Very Severe) | 1.94 (1.54-2.46) |  | 1.83 (1.32-2.54) * |  |  |
| Missing/Unknown | - |  | - |  |  |
| MRC dyspnoea score |  |  |  |  |  |
| 1-2 | 1.00 (ref) |  |  | 1.00 (ref) |  |
| 3 | 1.81 (1.50-2.18) | - | - | 1.53 (1.18-1.98) *p=0.001 | - |
| 4-5 | 2.99 (2.44-3.66) |  |  | 1.87 (1.42-2.46) * |  |
| Missing/Unknown | - |  |  | - |  |
| **Prior prevalent CVD** |  |  |  |  |  |
| Acute Coronary Syndrome | 2.37 (2.86-6.61) * | 0.99 (0.75-1.30) p=0.92 | 1.02 (0.72-1.45) p=0.92 | 0.95 (0.69-1.30) p=0.76 | 1.08 (0.80-1.47) p=0.60 |
| Arrhythmias | 2.70 (2.29-3.18) * | 1.40 (1.07-1.83) *p=0.014 | 1.53 (1.09-2.14) *p=0.013 | 1.40 (1.03-1.89) *p=0.032 | 1.34 (0.99-1.81) p=0.061 |
| Heart Failure | 7.00 (5.07-9.68) * | 2.57 (1.73-3.83) * | 2.80 (1.70-4.60) * | 2.99 (1.89-4.74) * | 2.67 (1.72-4.14) * |
| Pulmonary Hypertension | 2.97 (1.80-4.91) * | 0.90 (0.49-1.68) p=0.75 | 0.61 (0.28-1.34) p=0.22 | 1.17 (0.58-2.37) p=0.67 | 1.01 (0.51-2.00) p=0.99 |
| Ischaemic Stroke | 1.45 (1.16-1.82) *p=0.001 | 1.04 (0.76-1.43) p=0.82 | 0.95 (0.65-1.39) p=0.78 | 1.08 (0.74-1.56) p=0.69 | 1.09 (0.76-1.54) p=0.65 |
| **COPD Medications** |  |  |  |  |  |
| ***Long-acting, inhaled therapies*** |  |  |  |  |  |
| No long-acting therapies | 1.00 (ref) | 1.00 (ref) | 1.00 (ref) | 1.00 (ref) | 1.00 (ref) |
| LABA/LAMA mono/dual | 0.86 (0.68-1.09) p=0.21 | 0.84 (0.60-1.16) p=0.29 | 1.00 (0.66-1.50) p=0.98 | 0.75 (0.51-1.11) p=0.15 | 0.75 (0.52-1.09) p=0.13 |
| ICS-based mono/dual/triple | 0.96 (0.80-1.16) p=0.68 | 0.80 (0.61-1.04) p=0.10 | 0.96 (0.67-1.36) p=0.82 | 0.71 (0.51-0.97) *p=0.034 | 0.77 (0.56-1.04) p=0.084 |
| ***Short-acting, inhaled therapies*** |  |  |  |  |  |
| No short-acting therapies | 1.00 (ref) | 1.00 (ref) | 1.00 (ref) | 1.00 (ref) | 1.00 (ref) |
| SABA/SAMA mono/dual | 0.87 (0.71-1.05) p=0.15 | 0.83 (0.63-1.09) p=0.17 | 0.84 (0.61-1.15) p=0.27 | 0.80 (0.59-1.08) p=0.15 | 0.82 (0.61-1.11) p=0.19 |
| **CVD medications** |  |  |  |  |  |
| Positive inotropes | 4.10 (3.13-5.37) * | 1.52 (1.03-2.25) *p=0.033 | 1.43 (0.90-2.27) p=0.13 | 1.27 (0.82-1.97) p=0.28 | 1.35 (0.89-2.06) p=0.16 |
| Diuretics | 4.32 (3.72-5.01) * | 2.81 (2.29-3.45) * | 2.38 (1.86-3.05) * | 2.29 (1.82-2.88) * | 2.67 (2.13-3.36) * |
| Anti-arrhythmic drugs | 3.99 (2.66-5.98) * | 1.46 (0.87-2.45) p=0.15 | 1.83 (0.93-3.62) p=0.081 | 1.34 (0.74-2.41) p=0.33 | 1.27 (0.72-2.21) p=0.41 |
| Beta blockers | 3.07 (2.64-3.57) * | 1.65 (1.33-2.06) * | 1.33 (1.01-1.75) *p=0.04 | 1.64 (1.27-2.11) * | 1.57 (1.22-2.01) * |
| Hypertension and heart failure drugs | 2.41 (2.09-2.77) * | 1.43 (1.15-1.79) *p=0.002 | 1.41 (1.07-1.86) *p=0.016 | 1.39 (1.08-1.79) *p=0.010 | 1.52 (1.19-1.96) *p=0.001 |
| Nitrates, CCBs, other antianginals | 1.68 (1.47-1.92) * | 1.05 (0.86-1.29) p=0.64 | 0.97 (0.76-1.25) p=0.83 | 1.00 (0.79-1.26) p=0.99 | 0.98 (0.78-1.23) p=0.86 |
| Anticoagulants | 3.22 (2.69-3.85) * | 1.57 (1.16-2.13) *p=0.004 | 1.81 (1.24-2.63) *p=0.002 | 1.71 (1.21-2.40) *p=0.002 | 1.68 (1.19-2.37) *p=0.003 |
| Antiplatelets | 1.90 (1.65-2.18) * | 1.60 (1.28-2.00) * | 1.65 (1.25-2.17) * | 1.67 (1.29-2.16) * | 1.67 (1.30-2.14) * |
| Statins | 1.61 (1.41-1.85) * | 0.77 (0.61-0.96) *p=0.020 | 0.82 (0.63-1.08) p=0.16 | 0.76 (0.59-0.99) *p=0.039 | 0.78 (0.60-1.00) *p=0.049 |

eGFR = estimated glomerular filtration rate. uAlb = urine albumin. CVD=cardiovascular disease, ICS = inhaled corticosteroid, LABA = long-acting beta agonist, LAMA = long-acting muscarinic antagonist, SABA = short-acting beta agonist, SAMA = short-acting muscarinic antagonist, CCB = calcium channel blocker. n(%) described for the COPD and CV prescription categories may be mutually exclusive, as they can be taken in combination elsewhere in the table (e.g., patient prescribed a long-acting inhaler, short-acting inhaler, oral therapy, and a cardiovascular medication)

***** p < .001 unless otherwise stated.

The **minimally adjusted** model was adjusted for age and sex in all models, except age and sex where age was sex-adjusted, and sex was age-adjusted. N=4,699 for IMD, N=4,318 for BMI, N = 3,774 for CKD, N = 3,118 for GOLD, N = 3,641 for MRC because of missing data.

The **final model** was adjusted for socioeconomic status (IMD), smoking status, and comorbidities: BMI, Type II diabetes, depression and depressive symptoms, exacerbation severity, exacerbation frequency and prior prevalent CVD (hypertension, acute coronary syndrome, arrhythmia, heart failure, pulmonary hypertension, ischaemic stroke), and COPD medications (short-acting and long-acting COPD inhaled therapies) and major classes of CVD medications according to the British National Formulary (BNF).

**GOLD sensitivity analysis** model was the final model additionally adjusted for GOLD stage

**MRC sensitivity analysis** model was the final model additionally adjusted for MRC dyspoenea score

**CKD sensitivity analysis** model was the final model additionally adjusted for CKD

# Appendix 2. Supplementary Figures and Tables for Atrial Fibrillation

## Figure 2. Inclusion of patients in the case control study for atrial fibrillation/flutter


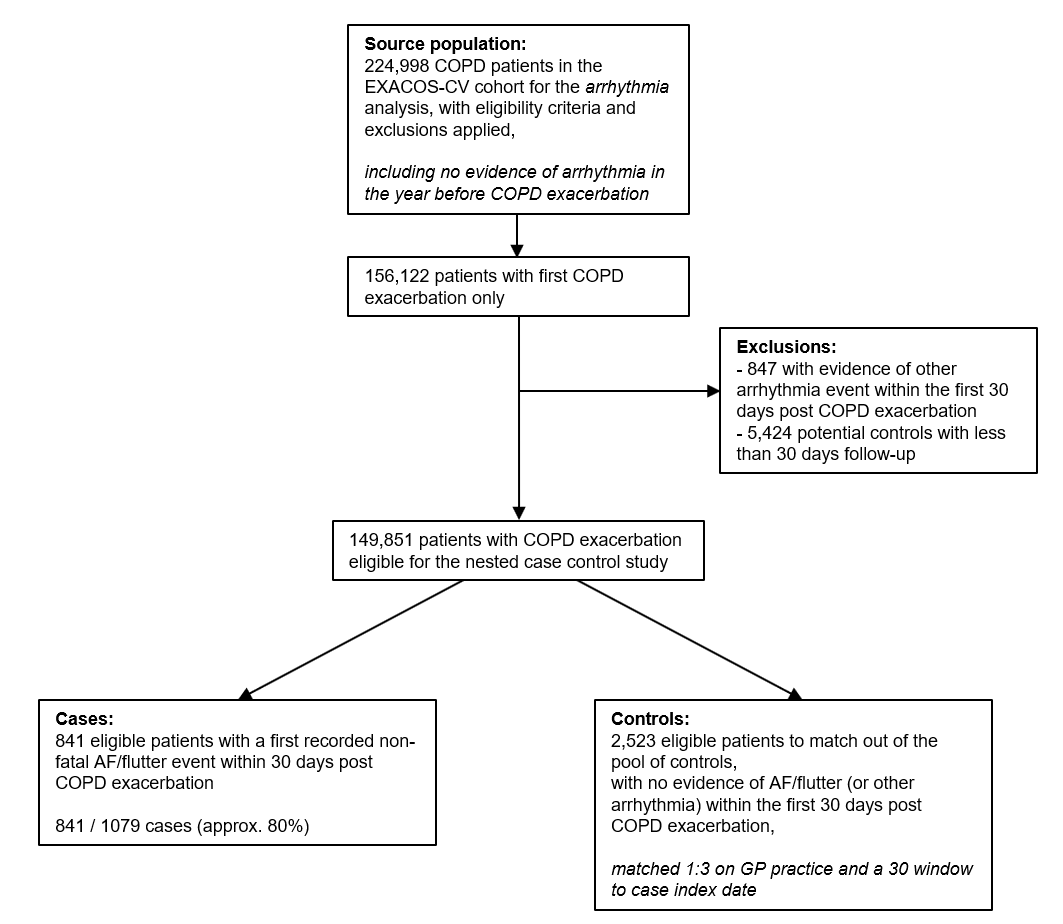


Legend: COPD (Chronic Obstructive Pulmonary Disease); GP (General Practitioner)

## Table 2. Factors associated with hospitalizations for atrial fibrillation or flutter within the first 30 days post acute exacerbation of COPD (minimally-adjusted models and sensitivity analyses)

| **Characteristic**  **N (%)** | **Minimally-adjusted**  **OR (95% CI)**  *Adjusted for age and sex*  (N=3,364 unless specified) | **Final Model**  **aOR (95% CI)**  (N=3,096) | **Sensitivity Analysis:**  **aOR (95% CI)**  *Final Model*  *+GOLD stage*  (N=2,259) | **Sensitivity Analysis**  **aOR (95% CI)**  *Final Model*  *+MRC dyspnea score*  (N=2,583) | **Sensitivity Analysis**  **aOR (95% CI)**  *Final Model*  *+CKD*  (N=2,474) |
| --- | --- | --- | --- | --- | --- |
| Age |  |  |  |  |  |
| 40-69 | 1.00 (ref) | 1.00 (ref) | 1.00 (ref) | 1.00 (ref) | 1.00 (ref) |
| 70-74 | 2.03 (1.55-2.67) * | 1.21 (0.85-1.73) p=0.30 | 1.45 (0.95-2.20) p=0.083 | 1.37 (0.92-2.04) p=0.118 | 1.04 (0.69-1.56) p=0.86 |
| 75-79 | 3.42 (2.66-4.40) * | 1.82 (1.28-2.58) *p=0.001 | 1.97 (1.30-2.99) *p=0.002 | 1.98 (1.35-2.91) *p=0.001 | 1.78 (1.21-2.61) *p=0.004 |
| 80+ | 6.31 (5.01-7.95) * | 3.15 (2.26-4.40) * | 3.32 (2.21-5.00) * | 3.19 (2.20-4.61) * | 2.74 (1.85-4.04) * |
| Male sex | 1.52 (1.28-1.81) * | 1.50 (1.18-1.91) *p=0.001 | 1.49 (1.11-1.99) *p=0.008 | 1.50 (1.15-1.95) *p=0.003 | 1.45 (1.12-1.89) *p=0.005 |
| Smoking status |  |  |  |  |  |
| Ex-smoker | 1.00 (ref) | 1.00 (ref) | 1.00 (ref) | 1.00 (ref) | 1.00 (ref) |
| Current smoker | 1.02 (0.85-1.23) p=0.82 | 1.17 (0.90-1.51) p=0.24 | 1.12 (0.83-1.51) p=0.46 | 1.22 (0.92-1.62) p=0.16 | 1.16 (0.87-1.55) p=0.31 |
| Index of Multiple Deprivation (IMD) quintile |  |  |  |  |  |
| Least Deprived | 1.00 (ref) | 1.00 (ref) | 1.00 (ref) | 1.00 (ref) | 1.00 (ref) |
| 2 | 1.06 (0.78-1.43) p=0.73 | 1.35 (0.88-2.06) p=0.17 | 1.34 (0.82-2.19) p=0.24 | 1.22 (0.77-1.94) p=0.40 | 1.33 (0.83-2.14) p=0.24 |
| 3 | 0.91 (0.66-1.26) p=0.58 | 0.98 (0.63-1.53) p=0.94 | 0.88 (0.52-1.50) p=0.65 | 0.88 (0.54-1.44) p=0.61 | 1.07 (0.64-1.76) p=0.81 |
| 4 | 0.68 (0.48-0.96) *p=0.027 | 0.81 (0.51-1.28) p=0.36 | 0.87 (0.51-1.48) p=0.61 | 0.66 (0.40-1.09) p=0.11 | 0.92 (0.55-1.52) p=0.74 |
| Most Deprived | 0.92 (0.65-1.31) p=0.65 | 1.10 (0.68-1.79) p=0.71 | 1.02 (0.57-1.83) p=0.94 | 0.95 (0.56-1.62) p=0.85 | 1.15 (0.67-1.98) p=0.62 |
| Ethnicity |  |  |  |  |  |
| White |  |  |  |  |  |
| South Asian |  |  |  |  |  |
| Black | - | - | - | - | - |
| Other |  |  |  |  |  |
| Mixed |  |  |  |  |  |
| Missing |  |  |  |  |  |
| **Comorbidities** |  |  |  |  |  |
| Type II Diabetes | 1.48 (1.21-1.81) * | 1.02 (0.76-1.37) p=0.89 | 1.13 (0.80-1.61) p=0.50 | 1.04 (0.76-1.44) p=0.79 | 0.97 (0.71-1.34) p=0.87 |
| Depression / depressive symptoms | 1.41 (1.15-1.74) *p=0.001 | 1.04 (0.76-1.41) p=0.82 | 1.06 (0.73-1.53) p=0.75 | 1.02 (0.72-1.43) p=0.92 | 0.98 (0.69-1.38) p=0.69 |
| Anxiety | 1.01 (0.82-1.24) p=0.93 | 1.04 (0.78-1.39) p=0.79 | 1.13 (0.80-1.59) p=0.49 | 1.04 (0.76-1.43) p=0.81 | 1.01 (0.73-1.39) p=0.95 |
| Hypertension | 1.43 (1.19-1.71) * | 1.03 (0.77-1.37) p=0.85 | 0.97 (0.69-1.36) p=0.86 | 1.10 (0.81-1.52) p=0.54 | 1.04 (0.76-1.42) p=0.80 |
| BMI |  |  |  |  |  |
| Normal (18.5 to <25) | 1.00 (ref) | 1.00 (ref) | 1.00 (ref) | 1.00 (ref) | 1.00 (ref) |
| Underweight (<18.5) | 1.00 (0.67-1.51) p=0.67 | 1.16 (0.69-1.94) p=0.58 | 1.23 (0.65-2.33) p=0.53 | 1.12 (0.62-2.02) p=0.71 | 1.35 (0.73-2.51) p=0.34 |
| Overweight (25 to <30) | 0.94 (0.75-1.16) p=0.75 | 0.94 (0.70-1.26) p=0.67 | 0.84 (0.59-1.19) p=0.32 | 0.92 (0.67-1.27) p=0.61 | 0.93 (0.67-1.30) p=0.67 |
| Obese (30+) | 1.00 (0.80-1.25) p=0.80 | 0.97 (0.71-1.32) p=0.85 | 0.89 (0.62-1.29) p=0.55 | 0.95 (0.68-1.33) p=0.76 | 0.88 (0.62-1.24) p=0.45 |
| Missing/Unknown | - | - | - | - | - |
| Chronic Kidney Disease |  |  |  |  |  |
| No (eGFR >60% or uAlb<3mg/mmol) | 1.00 (ref) |  |  |  | 1.00 (ref) |
| Yes | 1.84 (1.48-2.29) * | - | - | - | 1.23 (0.90-1.67) p=0.19 |
| Missing | - |  |  |  | - |
| **COPD prognosis** |  |  |  |  |  |
| Exacerbation severity  at start of follow-up |  |  |  |  |  |
| Moderate | 1.00 (ref) | 1.00 (ref) | 1.00 (ref) | 1.00 (ref) | 1.00 (ref) |
| Severe | 4.17 (3.43-5.06) * | 5.78 (4.45-7.50) * | 6.34 (4.61-8.71) * | 5.40 (4.05-7.19) * | 6.03 (4.50-8.10) * |
| Exacerbation frequency  in the 1-year window preceding 1-year-to-start of cohort follow-up |  |  |  |  |  |
| Infrequent exacerbator (≤1) | 1.00 (ref) | 1.00 (ref) | 1.00 (ref) | 1.00 (ref) | 1.00 (ref) |
| Frequent exacerbator (≥2) | 1.07 (0.86-1.33) p=0.53 | 0.81 (0.60-1.10) p=0.18 | 0.93 (0.65-1.32) p=0.68 | 0.81 (0.58-1.13) p=0.22 | 0.80 (0.57-1.12) p=0.19 |
| GOLD grade of airflow limitation |  |  |  |  |  |
| 1 (Mild) | 1.00 (ref) |  | 1.00 (ref) |  |  |
| 2 (Moderate) | 1.25 (0.96-1.62) p=0.10 | - | 1.07 (0.74-1.54) p=0.73 | - | - |
| 3-4 (Severe or Very Severe) | 1.52 (1.16-2.00) *p=0.003 |  | 1.20 (0.81-1.78) p=0.37 |  |  |
| Missing/Unknown | - |  | - |  |  |
| MRC dyspnoea score |  |  |  |  |  |
| 1-2 | 1.00 (ref) |  |  | 1.00 (ref) |  |
| 3 | 1.47 (1.17-1.85) *p=0.001 | - | - | 1.07 (0.78-1.47) p=0.65 | - |
| 4-5 | 2.24 (1.77-2.84) * |  |  | 1.31 (0.94-1.83) p=0.11 |  |
| Missing/Unknown | - |  |  | - |  |
| **Prior prevalent CVD** |  |  |  |  |  |
| Acute Coronary Syndrome | 1.81 (1.42-2.31) * | 0.76 (0.53-1.11) p=0.16 | 0.77 (0.49-1.21) p=0.25 | 0.76 (0.50-1.14) p=0.19 | 0.74 (0.49-1.10) p=0.14 |
| Arrhythmias | 7.90 (6.31-9.91) * | 3.55 (2.53-4.98) * | 3.85 (2.57-5.76) * | 3.42 (2.36-4.94) * | 3.53 (2.43-5.13) * |
| Heart Failure | 2.78 (1.91-4.05) * | 1.17 (0.70-1.98) p=0.55 | 1.21 (0.64-2.26) p=0.56 | 1.03 (0.57-1.85) p=0.92 | 1.11 (0.64-1.94) p=0.71 |
| Pulmonary Hypertension | 7.09 (3.47-14.5) * | 3.05 (1.21-7.68) *p=0.018 | 2.48 (0.81-7.59) p=0.11 | 3.43 (1.21-9.77) *p=0.021 | 3.33 (1.14-9.71) *p=0.028 |
| Ischaemic Stroke | 1.55 (1.16-2.06) *p=0.003 | 0.87 (0.57-1.32) p=0.51 | 0.75 (0.44-1.26) p=0.28 | 0.77 (0.48-1.23) p=0.28 | 0.94 (0.60-1.47) p=0.79 |
| **COPD Medications** |  |  |  |  |  |
| ***Long-acting, inhaled therapies*** |  |  |  |  |  |
| No long-acting therapies | 1.00 (ref) | 1.00 (ref) | 1.00 (ref) | 1.00 (ref) |  |
| LABA/LAMA mono/dual | 0.86 (0.61-1.22) p=0.40 | 1.14 (0.70-1.87) p=0.60 | 1.46 (0.79-2.69) p=0.23 | 1.17 (0.67-2.05) p=0.59 | 1.11 (0.64-1.91) p=0.71 |
| ICS-based mono/dual/triple | 1.06 (0.81-1.38) p=0.67 | 1.23 (0.85-1.79) p=0.28 | 1.53 (0.93-2.49) p=0.09 | 1.43 (0.91-2.24) p=0.13 | 1.10 (0.73-1.65) p=0.65 |
| ***Short-acting, inhaled therapies*** |  |  |  |  |  |
| No short-acting therapies | 1.00 (ref) | 1.00 (ref) | 1.00 (ref) | 1.00 (ref) |  |
| SABA/SAMA mono/dual | 0.95 (0.75-1.20) p=0.66 | 1.17 (0.84-1.62) p=0.35 | 1.16 (0.79-1.68) p=0.45 | 1.14 (0.81-1.62) p=0.45 | 1.11 (0.77-1.61) p=0.56 |
| **CVD medications** |  |  |  |  |  |
| Positive inotropes | 12.3 (8.39-17.9) * | 2.29 (1.41-3.74) *p=0.001 | 2.13 (1.17-3.87) *p=0.013 | 2.45 (1.41-4.25) *p=0.001 | 2.35 (1.39-3.97) *p=0.002 |
| Diuretics | 2.51 (2.10-3.00) * | 1.36 (1.04-1.77) *p=0.024 | 1.49 (1.08-2.07) *p=0.015 | 1.35 (1.00-1.81) p=0.050 | 1.43 (1.06-1.93) *p=0.019 |
| Anti-arrhythmic drugs | 6.78 (4.07-11.3) * | 2.14 (1.10-4.15) *p=0.025 | 2.47 (1.03-5.90) *p=0.043 | 2.56 (1.15-5.71) *p=0.022 | 2.05 (1.00-4.24) p=0.051 |
| Beta blockers | 3.12 (2.56-3.79) * | 1.52 (1.14-2.03) *p=0.004 | 1.54 (1.09-2.18) *p=0.014 | 1.66 (1.20-2.29) *p=0.002 | 1.53 (1.12-2.08) *p=0.008 |
| Hypertension and HF drugs | 1.66 (1.40-1.98) * | 0.91 (0.69-1.21) p=0.52 | 0.85 (0.61-1.19) p=0.35 | 0.91 (0.67-1.24) p=0.56 | 0.87 (0.64-1.18) p=0.36 |
| Nitrates, CCBs, other antianginals | 1.45 (1.22-1.73) * | 1.04 (0.80-1.34) p=0.77 | 1.22 (0.90-1.66) p=0.19 | 1.00 (0.76-1.32) p=0.99 | 1.02 (0.77-1.35) p=0.90 |
| Anticoagulants | 8.16 (6.36-10.5) * | 3.81 (2.57-5.64) * | 4.52 (2.80-7.29) * | 3.82 (2.45-5.96) * | 3.56 (2.33-5.44) * |
| Antiplatelets | 1.37 (1.15-1.62) * | 1.46 (1.10-1.93) *p=0.008 | 1.38 (1.00-1.92) p=0.053 | 1.31 (0.96-1.78) p=0.089 | 1.46 (1.07-1.98) *p=0.016 |
| Statins | 1.52 (1.27-1.81) * | 0.95 (0.72-1.26) p=0.722 | 0.95 (0.68-1.33) p=0.78 | 0.96 (0.70-1.31) p=0.80 | 1.00 (0.73-1.38) p=0.98 |

eGFR = estimated glomerular filtration rate. uAlb = urine albumin. CVD=cardiovascular disease, ICS = inhaled corticosteroid, LABA = long-acting beta agonist, LAMA = long-acting muscarinic antagonist, SABA = short-acting beta agonist, SAMA = short-acting muscarinic antagonist, CCB = calcium channel blocker. n(%) described for the COPD and CV prescription categories may be mutually exclusive, as they can be taken in combination elsewhere in the table (e.g., patient prescribed a long-acting inhaler, short-acting inhaler, oral therapy, and a cardiovascular medication)

***** p < .001 unless otherwise stated.

The **minimally adjusted** model was adjusted for age and sex in all models, except age and sex where age was sex-adjusted, and sex was age-adjusted. N=3,096 for BMI, N = 2,663 for CKD, N = 2,371 for GOLD, N = 2,740 for MRC because of missing data.

The **final model** was adjusted for socioeconomic status (IMD), smoking status, and comorbidities: BMI, Type II diabetes, depression and depressive symptoms, exacerbation severity, exacerbation frequency and prior prevalent CVD (hypertension, acute coronary syndrome, arrhythmia, heart failure, pulmonary hypertension, ischaemic stroke), and COPD medications (short-acting and long-acting COPD inhaled therapies) and major classes of CVD medications according to the British National Formulary (BNF).

**GOLD sensitivity analysis** model was the final model additionally adjusted for GOLD stage

**MRC sensitivity analysis** model was the final model additionally adjusted for MRC dyspoenea score

**CKD sensitivity analysis** model was the final model additionally adjusted for CKD

# Appendix 3. Extended Methods

## Table 3. Extended case definitions

| **Outcome** | **Outcome includes the following phenotypes:** |
| --- | --- |
| **Atrial Fibrillation / Flutter** | Atrial fibrillation  Atrial flutter  (No other arrhythmias) |
| **Heart Failure** | HFpEF  HFrEF  Decompensated HF  Acute pulmonary oedema  Dilated cardiomyopathy  Takotsubo cardiomyopathy |

Cases defined as hospitalised cardiovascular events as registered in linked HES data using ICD-10 codes for admission. A full list of codes (codelists) for each variable are available on our EXACOS-CV [GitHub repository](https://github.com/NHLI-Respiratory-Epi/codelists_EXACOS-CV). Methods to create our codelists used our standardisable, reproducible methodology,(1) available on our GitHub repository: for [drug codelists](https://github.com/NHLI-Respiratory-Epi/drug-codelist-creation) and for [medical/phenotype codelists](https://github.com/NHLI-Respiratory-Epi/SNOMED-CT-codelists), respectively.

## Table 4. Extended covariate definitions

| **Covariate** | **Categorization** | **Period of measurement/patient history before exacerbation date (cohort index date); additional information** |
| --- | --- | --- |
| **Patient-related** |  |  |
| Age | Categorical | Cohort index date   - 40-69 - 70-74 - 75-79 - 80+   Younger age categories combined due to data sparsity (40-69) |
| Sex | Male / female | Cohort index date |
| Socioeconomic status, using Index of Multiple Deprivation (IMD) Scores for England | Quintile | Cohort index date  The deprivation score is derived for 32,482 areas in the UK, ranging from 1 (most deprived) to 32,482 (least deprived). CPRD links this deprivation score and can supply the scores that split the areas into 5 groups (i.e., quintiles) of decreasing deprivation.  *Linked data.* |
| Ethnicity | Quintile | Cohort index date  Categories: White, South Asian, Black, Mixed, Other as defined  ‘Unstated’ ethnicity was considered to be ‘missing.’  Variable categorisation based on previous methods.(2) |
| **Comorbidities** |  |  |
| Smoking history | Past/present | Most recent in patient history.  2 categories: ex-smoker, current smoker |
| BMI | Categorical (5) | Last 5 years of patient history (measurement closest to cohort index date was selected).  Weight in kilograms divided by height in meters squared, will be utilized as either a continuous variable or a categorical variable using the following categories widely referenced as WHO classification: Underweight (Below 18.5), Normal (18.5 - 24.9), Overweight (25.0 - 29.9), and Obese (30.0 and greater). |
| Type II diabetes | Yes/no | Anytime preceding cohort index date  Variable categorisation was based on previous methodology.(3) |
| Current asthma | Yes/no | A diagnosis of asthma (=1) vs. no history of asthma (=0) in a period of 3 years before a 2-year period prior to COPD diagnosis.  This is because asthma diagnoses made 2 years prior to a COPD diagnosis is likely to be misclassified COPD. Any asthma diagnosis made 3 years prior to this is therefore likely to be a current asthma diagnosis.  This definition is based on previous methods.(4) |
| Chronic kidney disease | Yes/no | 24-month period preceding cohort index date  A diagnosis of CKD (=1) vs. no diagnosis (=0), the diagnoses  identified through either:   - an estimated glomerular filtration rate (eGFR) of <60 mL/min/1.73 m2 (i.e., <60% kidney function)   *or*   - a urine albumin:creatinine ratio (uACR) of >3mg/mmol (i.e., indicating proteinuria)   This algorithm is based on previous methods(5) and is based on an study assessing the use of both eGFR and uACR measurements to define CKD.(6) |
| Depression and depressive symptoms | Yes/no | Anytime preceding cohort index date |
| Anxiety | Yes/no | Anytime preceding cohort index date |
| Hypertension | Yes/no | Anytime preceding cohort index date |
| Prior prevalent cardiovascular disease   - Acute Coronary Syndrome - Arrhythmias - Heart failure - Ischaemic stroke - Pulmonary hypertension | Yes/no  for each | Anytime preceding one year before cohort index date, defined through primary-care records, to be distinguished with the outcomes defined through hospital records.  This is because we excluded patients if they had evidence of prior incident CV event in the year window before cohort index date.  Refer to extended description of cases for full definitions of encompassed phenotypes |
| **Medication** |  |  |
| Respiratory medication for COPD – short acting inhalers | Yes/No | 24-month period preceding cohort index date   - No (ref.) - Yes   SABA  SAMA  SABA/SAMA fixed/open combinations  Mono/dual therapies combined to reduce multiplicity |
| Respiratory medication for COPD – long acting inhalers | Categorical (3) | 24-month period preceding cohort index date   - No (ref.) - LABA/LAMA mono/dual fixed/open (non-ICS) - ICS-based   ICS/LABA fixed/open  ICS/LAMA fixed/open  ICS/LABA+LAMA fixed and open (triple)  ICS-based combined; non-ICS combined – to reduce multiplicity |
| Cardiovascular medication,  covering certain drug classes of [British National Formulary Chapter 2](https://openprescribing.net/bnf/02/)   - 2.1 Positive inotropic drugs - 2.2 Diuretics - 2.3 Anti-arrhythmic drugs - 2.4 Beta blockers - 2.5 Hypertension and heart failure drugs - 2.6 Nitrates, calcium-channel blockers & other antianginal drugs - 2.8 Anticoagulants - 2.9 Antiplatelets - 2.12 Statins | Yes/no  for each | 24-month period preceding cohort index date  All medications are of oral route given as what is most commonly prescribed in routine care, except for 2.6 (oral, parenteral, transdermal) and 2.8 (oral and parenteral). |
| **Other covariates indicating COPD progression** |  |  |
| Medical Research Council (MRC) dyspnoea scale measurement | Categorical | Within 24-month period preceding cohort index date   - Category 1 (ref.)   Grade 1: No Dyspnea except on strenuous Exercise  Grade 2: Short of breath when walking up a short hill   - Category 2   Grade 3: Dyspnea limits walking pace (slower than others) and stops to catch breath   - Category 3   Grade 4: Stops to catch breath after walking 100 meters (328 feet) on level ground  Grade 5: Dyspnea prevents leaving house and performing Activities of Daily Living   - Missing / Unknown / Unavailable if no record available within the 24 month prior to and up  to 3 months following index date |
| Lung function: COPD GOLD grade of airflow limitation | Categorical | Within 24 months prior to 3 months after cohort index date for exacerbation, defined according to GOLD-defined lung obstruction-based classification:   - Category 1 (ref.)   GOLD 1: FEV_1_% predicted ≥80% (Mild)   - Category 2   GOLD 2: FEV_1_ % predicted ≥50 -<80% (Moderate)   - Category 3   GOLD 3: FEV_1_% predicted ≥30 -<50% (Severe)  GOLD 4: FEV_1_ % predicted <30% (Very Severe)   - Missing/ Unknown (if missing spirometry during the 24 months prior to 3 months after cohort index date) |
| Cumulative number of prior exacerbations | Categorical | 12 month window in the period before the 12 months-to-cohort index date.   - 0-1 exacerbations (Infrequent) (ref.) - 2+ exacerbations (Frequent)   Categories combined to reduce multiplicity. Categories based on previous case-control study.(7)  *Encompasses both moderate and severe exacerbations, i.e., from primary care (GP) and linked secondary care data (HES), respectively.* |

All covariates unless stated based on CPRD primary care data. A full list of codes (codelists) for each variable are available on our EXACOS-CV [GitHub repository](https://github.com/NHLI-Respiratory-Epi/codelists_EXACOS-CV). Methods to create our codelists used our standardisable, reproducible methodology,(1) available on our GitHub repository: for [drug codelists](https://github.com/NHLI-Respiratory-Epi/drug-codelist-creation) and for [medical/phenotype codelists](https://github.com/NHLI-Respiratory-Epi/SNOMED-CT-codelists), respectively.

## Power calculations

Given a small proportion of patients experience an acute cardiovascular event immediately following an exacerbation, we carried out sample power calculations to verify the study would have sufficient power to detect significant differences in the odds of experiencing a cardiovascular event for a given factor, relative to the factor’s reference group.

For example, the prevalence of Type II diabetes among exacerbating, COPD patients was 19.3% in the EXACOS-CV study.(8) Taking this prevalence into account, for HF, for a 1:2 matching of cases and controls, with 1,569 cases and 3,138 controls, a power of 80%, and an α of 5% (type 1 error), the minimum and maximum detectable ORs would be 0.796 (highest OR<1) and 1.236 (smallest OR >1), respectively. For AF/flutter, for a 1:3 matching of cases and controls, with 841 cases and 2,523 controls, the minimum and maximum detectable ORs would be 0.741 (highest OR<1) and 1.309 (smallest OR >1), respectively.

# STROBE and RECORD Checklist

## Table 5. STROBE and RECORD Checklist(9)

|  | **Item No.** | **STROBE items** | **Location in manuscript where items are reported** | **RECORD items** | **Location in manuscript where items are reported** |
| --- | --- | --- | --- | --- | --- |
| **Title and abstract** | | | | | |
|  | 1 | (a) Indicate the study’s design with a commonly used term in the title or the abstract (b) Provide in the abstract an informative and balanced summary of what was done and what was found | Title (study design)  Abstract (study design) | RECORD 1.1: The type of data used should be specified in the title or abstract. When possible, the name of the databases used should be included.  RECORD 1.2: If applicable, the geographic region and timeframe within which the study took place should be reported in the title or abstract.  RECORD 1.3: If linkage between databases was conducted for the study, this should be clearly stated in the title or abstract. | Abstract (data type and database names)  Abstract (geographic  region and timeframe)  Abstract (linkage  specified) |
| **Introduction** | | | | | |
| Background rationale | 2 | Explain the scientific background and rationale for the investigation being reported | Introduction |  | Introduction |
| Objectives | 3 | State specific objectives, including any prespecified hypotheses | Introduction |  | Introduction |
| **Methods** | | | | | |
| Study Design | 4 | Present key elements of study design early in the paper | Methods |  | Methods |
| Setting | 5 | Describe the setting, locations, and relevant dates, including periods of recruitment, exposure, follow-up, and data collection | Methods |  | Methods |
| Participants | 6 | *(a) Cohort study* - Give the eligibility criteria, and the sources and methods of selection of participants. Describe methods of follow-up  *Case-control study* - Give the eligibility criteria, and the sources and methods of case ascertainment and control selection. Give the rationale for the choice of cases and controls  *Cross-sectional study* - Give the eligibility criteria, and the sources and methods of selection of participants  *(b) Cohort study* - For matched studies, give matching criteria and number of exposed and unexposed  *Case-control study* - For matched studies, give matching criteria and the number of controls per case | a. Methods  b. Methods, Results | RECORD 6.1: The methods of study population selection (such as codes or algorithms used to identify subjects) should be listed in detail. If this is not possible, an explanation should be provided.  RECORD 6.2: Any validation studies of the codes or algorithms used to select the population should be referenced. If validation was conducted for this study and not published elsewhere, detailed methods and results should be provided.  RECORD 6.3: If the study involved linkage of databases, consider use of a flow diagram or other graphical display to demonstrate the data linkage process, including the number of individuals with linked data at each stage. | 6.1. Codelists are publicly available on [Github](https://github.com/NHLI-Respiratory-Epi/codelists_EXACOS-CV). Codelists used our standardizable, reproducible methodology for [drugs](https://github.com/NHLI-Respiratory-Epi/drug-codelist-creation) and [phenotypes](https://github.com/NHLI-Respiratory-Epi/SNOMED-CT-codelists),. All codelists checked by clinicians. Codelists/algorithms previously tested.  6.2. Validation studies  cited where relevant,  and detailed methods for generating new codelists based on publically-available algorithms (for medical conditions and prescriptions.)  6.3. Figures S1, S2 |
| Variables | 7 | Clearly define all outcomes, exposures, predictors, potential confounders, and effect modifiers. Give diagnostic criteria, if applicable. | Methods; supplementary material extended methods | RECORD 7.1: A complete list of codes and algorithms used to classify exposures, outcomes, confounders, and effect modifiers should be provided. If these cannot be reported, an explanation should be provided. | 7.1 See 6.1-6.2 |
| Data sources/ measurement | 8 | For each variable of interest, give sources of data and details of methods of assessment (measurement).  Describe comparability of assessment methods if there is more than one group | Methods; supplementary material extended methods |  | Methods; supplementary material extended methods |
| Bias | 9 | Describe any efforts to address potential sources of bias | Methods; Discussion |  | Methods; Discussion |
| Study size | 10 | Explain how the study size was arrived at | Methods; Figures S1, S2 |  | Methods; Figures S1, S2 |
| Quantitative variables | 11 | Explain how quantitative variables were handled in the analyses. If applicable, describe which groupings were chosen, and why | Methods; supplementary material extended methods |  | Methods; supplementary material extended methods |
| Statistical methods | 12 | (a) Describe all statistical methods, including those used to control for confounding  (b) Describe any methods used to examine subgroups and interactions  (c) Explain how missing data were addressed  (d) *Cohort study* - If applicable, explain how loss to follow-up was addressed  *Case-control study* - If applicable, explain how matching of cases and controls was addressed  *Cross-sectional study* - If applicable, describe analytical methods taking account of sampling strategy  (e) Describe any sensitivity analyses | a-c. Methods  d. Methods; supplementary material extended methods  e. Methods (sensitivity analyses) |  | a-c. Methods  d. Methods; supplementary material extended methods  e. Methods (sensitivity analyses) |
| Data access and cleaning methods |  | .. |  | RECORD 12.1: Authors should describe the extent to which the investigators had access to the database population used to create the study population.  RECORD 12.2: Authors should provide information on the data cleaning methods used in the study. | Methods, Data Sharing Statement |
| Linkage |  | .. |  | RECORD 12.3: State whether the study included person-level, institutional-level, or other data linkage across two or more databases. The methods of linkage and methods of linkage quality evaluation should be provided. | Methods |
| **Results** | | | | | |
| Participants | 13 | (a) Report the numbers of individuals at each stage of the study (*e.g.*, numbers potentially eligible, examined for eligibility, confirmed eligible, included in the study, completing follow-up, and analysed)  (b) Give reasons for non-participation at each stage.  (c) Consider use of a flow diagram | (a) Figures S1, S2  (b) Methods, specifically rational for matching, and Limitations section on matching | RECORD 13.1: Describe in detail the selection of the persons included in the study (*i.e.,* study population selection) including filtering based on data quality, data availability and linkage. The selection of included persons can be described in the text and/or by means of the study flow diagram. | (a) Figures S1, S2; Methods  (b) Methods, specifically rational for matching, and Limitations section on matching |
| Descriptive data | 14 | (a) Give characteristics of study participants (*e.g.*, demographic, clinical, social) and information on exposures and potential confounders  (b) Indicate the number of participants with missing data for each variable of interest  (c) *Cohort study* - summarise follow-up time (*e.g.*, average and total amount) | (a, b) results and Tables 1 and 2 |  | (a, b) results and Tables 1 and 2 |
| Outcome data | 15 | *Cohort study* - Report numbers of outcome events or summary measures over time  *Case-control study* - Report numbers in each exposure category, or summary measures of exposure  *Cross-sectional study* - Report numbers of outcome events or summary measures | Results and main tables, and Supplementary figures and tables |  | Results and main tables, and Supplementary figures and tables |
| Main results | 16 | (a) Give unadjusted estimates and, if applicable, confounder-adjusted estimates and their precision (e.g., 95% confidence interval). Make clear which confounders were adjusted for and why they were included  (b) Report category boundaries when continuous variables were categorized  (c) If relevant, consider translating estimates of relative risk into absolute risk for a meaningful time period | (a) Minimally adjusted estimates in supplement, final model in main results, sensitivity analyses in supplement  (b) Results and supplement  (c) N/A |  | (a) Minimally adjusted estimates in supplement, final model in main results, sensitivity analyses in supplement  (b) Results and supplement  (c) N/A |
| Other analyses | 17 | Report other analyses done—e.g., analyses of subgroups and interactions, and sensitivity analyses | Results and Supplementary Materials |  | Results and Supplementary Materials |
| **Discussion** | | | | | |
| Key results | 18 | Summarise key results with reference to study objectives | Discussion |  | Discussion |
| Limitations | 19 | Discuss limitations of the study, taking into account sources of potential bias or imprecision. Discuss both direction and magnitude of any potential bias | Discussion | RECORD 19.1: Discuss the implications of using data that were not created or collected to answer the specific research question(s). Include discussion of misclassification bias, unmeasured confounding, missing data, and changing eligibility over time, as they pertain to the study being reported. | Discussion |
| Interpretation | 20 | Give a cautious overall interpretation of results considering objectives, limitations, multiplicity of analyses, results from similar studies, and other relevant evidence | Discussion |  | Discussion |
| Generalisability | 21 | Discuss the generalisability (external validity) of the study results | Discussion |  | Discussion |
| **Other Information** | | | | | |
| Funding | 22 | Give the source of funding and the role of the funders for the present study and, if applicable, for the original study on which the present article is based | Administrative section (funding) + Introduction |  | Administrative section (funding) + Introduction |
| Accessibility of protocol, raw data, and programming code |  | .. | Methods (software and reproducibility) + Administrative section (protocol number) | RECORD 22.1: Authors should provide information on how to access any supplemental information such as the study protocol, raw data, or programming code. | Methods (software and reproducibility) + Administrative section (protocol number) |

Checklist protected under Creative Commons Attribution ([CC BY](https://creativecommons.org/licenses/by/4.0/)) license

# References for Supplementary Materials

1. Graul EL, Stone PW, Massen GM, Hatam S, Adamson A, Denaxas S, et al. Determining prescriptions in electronic healthcare record data: methods for development of standardized, reproducible drug codelists. JAMIA Open [Internet]. 2023 Jul 4 [cited 2023 Sep 1];6(3):ooad078. Available from: https://academic.oup.com/jamiaopen/article/doi/10.1093/jamiaopen/ooad078/7252957

2. Mathur R, Bhaskaran K, Chaturvedi N, Leon DA, vanStaa T, Grundy E, et al. Completeness and usability of ethnicity data in UK-based primary care and hospital databases. J Public Health [Internet]. 2014 Dec [cited 2022 Apr 27];36(4):684–92. Available from: https://academic.oup.com/jpubhealth/article-lookup/doi/10.1093/pubmed/fdt116

3. Farmer R, Mathur R, Bhaskaran K, Eastwood SV, Chaturvedi N, Smeeth L. Promises and pitfalls of electronic health record analysis. Diabetologia. 2018 Jun;61(6):1241–8.

4. Nissen F, Morales DR, Mullerova H, Smeeth L, Douglas IJ, Quint JK. Concomitant diagnosis of asthma and COPD: a quantitative study in UK primary care. Br J Gen Pract [Internet]. 2018 Nov [cited 2022 Nov 2];68(676):e775–82. Available from: https://bjgp.org/lookup/doi/10.3399/bjgp18X699389

5. Cook S, Schmedt N, Broughton J, Kalra PA, Tomlinson LA, Quint JK. Characterising the burden of chronic kidney disease among people with type 2 diabetes in England: a cohort study using the Clinical Practice Research Datalink. BMJ Open [Internet]. 2023 Mar [cited 2023 Mar 22];13(3):e065927. Available from: https://doi.org/10.1136/bmjopen-2022-065927

6. Chronic Kidney Disease Prognosis Consortium. Association of estimated glomerular filtration rate and albuminuria with all-cause and cardiovascular mortality in general population cohorts: a collaborative meta-analysis. The Lancet [Internet]. 2010 Jun 12 [cited 2023 Jan 23];375(9731):2073–81. Available from: https://doi.org/10.1016/S0140-6736(10)60674-5

7. Quint J, Windsor C, Herrett E, Smeeth L. No association between exacerbation frequency and stroke in patients with COPD. Int J Chron Obstruct Pulmon Dis [Internet]. 2016 Feb;217. Available from: https://www.dovepress.com/no-association-between-exacerbation-frequency-and-stroke-in-patients-w-peer-reviewed-article-COPD

8. Graul EL, Nordon C, Rhodes K, Marshall J, Menon S, Kallis C, et al. Temporal risk of non-fatal cardiovascular events post COPD exacerbation: a population-based study [Accepted 20 December 2023]. Am J Respir Crit Care Med [Internet]. 2023 Dec; Available from: https://doi.org/10.1164/rccm.202307-1122OC

9. Benchimol EI, Smeeth L, Guttmann A, Harron K, Moher D, Petersen I, et al. The REporting of studies Conducted using Observational Routinely-collected health Data (RECORD) Statement. PLOS Med [Internet]. 2015 Oct 6 [cited 2023 Jan 1];12(10):e1001885. Available from: https://doi.org/10.1371/journal.pmed.1001885

# References for Datasets (CPRD and Linked)

1. Clinical Practice Research Datalink. (2022). **CPRD Aurum** May 2022 (Version 2022.05.001) [Data set]. Clinical Practice Research Datalink. <https://doi.org/10.48329/t89s-kf12>
2. Clinical Practice Research Datalink. (2022). CPRD Aurum **Small Area data (patient)** January 2022 (Version 2022.01.001) [Data set]. Clinical Practice Research Datalink. <https://doi.org/10.48329/AYTT-H222>
3. Clinical Practice Research Datalink. (2022). CPRD Aurum **HES APC** January 2022 (Version 2022.01.001) [Data set]. Clinical Practice Research Datalink. <https://doi.org/10.48329/VAGX-9D96>
4. Clinical Practice Research Datalink. (2022). CPRD Aurum **ONS** deaths January 2022 (Version 2022.01.001) [Data set]. Clinical Practice Research Datalink. <https://doi.org/10.48329/Q34F-F505>
